# A Ni‐Bonded Hydride in Superatomic Silver Nanoclusters: Probing Synergistic Heteroatomic Core Effects on Oxygen Evolution Reactivity

**DOI:** 10.1002/smll.202512495

**Published:** 2025-11-11

**Authors:** Yu‐Rong Ni, Tzu‐Hao Chiu, Rugma T P, Michael N. Pillay, Samia Kahlal, Jean‐Yves Saillard, C. W. Liu

**Affiliations:** ^1^ Department of Chemistry National Dong Hwa University No. 1, Sec 2, Da Hsueh Rd. Hualien 97401 Taiwan; ^2^ CNRS, ISCR‐UMR 6226 Univ Rennes Rennes F‐35000 France

**Keywords:** heteroatom doping, hydride, oxygen evolution reaction, superatom

## Abstract

In the context of the intensely investigated field of heterometal doping of coinage nanoclusters (NCs), an increasing attention has shifted toward NCs doped with first‐row transition metals due to their economic viability. Herein, the Ni‐Ag bimetallic NCs [NiAg_20_{S_2_P(O*^i^* Pr)_2_}_12_] **NiAg_20_** and [NiHAg_19_{S_2_P(O*^i^* Pr)_2_}_12_] **NiHAg_19_** are reported, which are synthesized via a one‐pot reduction method. **NiHAg_19_** is the first example of an NC containing an encapsulated NiH unit and a rare species exhibiting Ni(0)─H bonding. The **NiAg_20_** and **NiHAg_19_** compositions are confirmed by single‐crystal X‐ray diffraction (SCXRD) and supported by density functional theory (DFT) calculations. Both NCs feature a superatomic 8‐electron count with 1S^2^1P^6^ configuration. The effect of nickel‐doping on the oxygen evolution reaction (OER) catalytic performance is investigated. It significantly enhances alkaline OER activity, with **NiAg_20_** and **NiHAg_19_** showing improved catalytic performances compared to their monometallic silver counterparts.

## Graphical abstract

The synthesis and detailed characterization of novel Ni‐Ag superatomic nanoclusters, including a unique Ni‐bonded hydride, with structure‐property investigation in the context of OER catalysis.

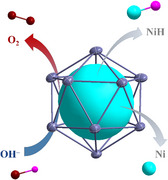

## 1 Introduction

Nanoclusters (NCs) are a prominent research topic due to their unique physical and chemical properties, which lead to distinctive functionalities and promising applications.^[^ ^1^, ^2^, ^3^, ^4^, ^5^, ^6^, ^7^ ^]^ Among these, heterometal‐doped NCs are particularly noteworthy. Their electronic structures often yield tunable photophysical properties and enhanced stability, leading to remarkable performance in catalytic processes.^[^ ^8^, ^9^, ^10^ ^]^ Recent studies have shown that coinage metal NCs doped with a single platinum‐group metal exhibit superior electrocatalytic efficiency in the hydrogen evolution reaction (HER), oxygen evolution reaction (OER), and CO_2_ reduction, owing to synergistic effects between the heterometal and the intrinsic properties of the NCs.^[^ ^11^, ^12^, ^13^, ^14^, ^15^ ^]^ Research on NC optimization by heterometal doping has historically focused on second‐row or heavier transition metals. However, there's a growing recognition of the untapped potential of cheaper, earth‐abundant first‐row transition metals, which can exhibit favorable synergistic effects. With respect to the first‐row metals, namely nickel, only a few examples of Ni‐doped species have been structurally characterized to date, such as [NiAg_24_(SPhMe_2_)_18_]^2−^ and [NiAu_24_(C≡CAr^F^)_18_].^2−[^ ^16^, ^17^, ^18^ ^]^ It is of note that in these two species, the Ni atom occupies the center of an icosahedral 8‐electron Ni@Au_12 _core. This position was also predicted by density functional theory (DFT) calculations on a series of 8‐electron [NiAg_20_(E_2_PH_2_)_12_] (E = S, Se) models with centered Ni@Ag_12_ icosahedral cores.^[^ ^19^ ^]^

Incorporating hydrides into coinage metal superatomic NCs introduces a fascinating duality in their chemical role. In the general case, hydrides behave as *classical* ligands, coordinating to the metallic core of the NC and located on its outer shell (NC surface). On the other hand, it has been shown recently that a hydrogen atom can also be encapsulated within the superatomic core of the NC. Strongly bonded to the central metal (thus forming encapsulated MH units with the central metal), it becomes an integral part of the superatomic core, with its electron contributing to the overall superatomic electron count.^[^ ^20^, ^21^, ^22^, ^23^, ^24^ ^]^ This distinction is essential because these two situations lead to different physicochemical properties for the superatoms. At first sight, determining the position of hydrides by single‐crystal X‐ray diffraction (SCXRD) in noble metal NCs appears very challenging owing to the hydrogen's low atomic number and its proximity to the metal atoms. However, the location of the encapsulated hydrides can be identified indirectly from the significant distortion they provoke on their metallic host. This information is completed by NMR spectroscopy, which is particularly valuable for probing hydride environments, as it reveals unique coupling effects and provides crucial information about their coordination. Furthermore, the experimental data can be confronted with DFT computational results, which, in addition to structural elucidation, allow understanding the hydride electronic participation to the superatomic system.

Among coinage metal NCs, several species exhibiting a central M or MH (M = Pd, Pt) unit have been isolated with varying shell configurations and properties. Au‐rich systems have been particularly prominent, with compounds of the type [PtHAu_8_(PPh_3_)^8^]^+^ reported early on,^[^ ^25^, ^26^, ^27^ ^]^ followed later by a PdH analogue isolated by Tsukuda and coworkers.^[^ ^27^ ^]^ The same group isolated heteroleptic MAu_24_L_18_ (M = Ni, Pd, Pt; L = alkynyl, thiolate), among which, the first Ni‐doped superatom [NiAu_24_(C≡CAr^F^)_18_].^2−18^ Crucially, the synthesis utilizes a crude precursor identified as [HNiAu_9_(PPh_3_)_8_Cl]^+^, which, unlike Pt and Pd analogues, has not been fully characterized.^[^ ^17^ ^]^ Lee and coworkers have developed the Ag‐rich system MAg_24_(SPhMe_2_)_18_ (M = Ni, Pd, Pt) by metal exchange to generate the NiAg_24_(SPhMe_2_)_18_ from a parent monometallic Ag_25_(SPhMe_2_)_18_.^[^ ^16^ ^]^

In superatomic and non‐superatomic copper‐rich NCs, the central metal atom can coordinate to one or more hydride(s), leading to the encapsulation of MH, MH_2_ (M = Pt/Pd)^[^ ^11^, ^12^, ^28^, ^29^ ^]^ and even PtH_3_^[^ ^30^ ^]^ units. With respect to silver‐rich systems, we have reported the first PtH‐encapsulating example, [PtHAg_19_(dtp)_12_] (dtp = dithiophosphate), which contains an icosahedral [(PtH)@Ag_12_]^5+^ 8‐electron superatomic core, in which the hydride is explicitly confirmed by Neutron diffraction to occupy a tetrahedral PtAg_3_ cavity.^[^ ^31^ ^]^ Later, its selenium homologue [PtHAg_19_(dsep)_12_] (dsep = diselenophosphate) was also isolated,^[^ ^31^ ^]^ as well as the palladium relatives [PdHAg_19_L_12_] and [PdHAg_20_L_12_]^+^ (L = dtp, dsep).^[^ ^32^, ^33^, ^34^ ^]^ With M = Rh and Ir, both MH and MH_2_ encapsulations in icosahedral 8‐electron systems have been reported: [(RhH)@Ag_20_(dtp/dsep)_12_],^[^ ^35^, ^36^ ^]^ [(RhH*_2_*)@Ag_19_(dtp/dsep)_12_]^[^ ^35^, ^36^ ^]^ and [IrHAg_24_(SR)_18_].^2−[^ ^37^ ^]^ In the case of Ru and Os, Lee and coworkers isolated the dihydride‐containing NCs [(MH*_2_*)Ag_24_(SR)_18_]^2−^ (R = Ru, Os).^[^ ^37^ ^]^ Finally, one should mention that we also developed 16‐electron species, formed from the vertex fusion of two 8‐electron icosahedral motifs, which can also retain hydrides within their superatomic core.^[^ ^38^ ^]^

It should be noted that in all the examples reported above, the encapsulated hydrogen is coordinated to a metal whose electronegativity is greater than (Rh and Pt; 2.28) or equal to (Ru, Os, Ir, Pd) that of hydrogen (2.20). This observation initially suggested that metals with a significantly lower electronegativity, such as Ni (1.91), might be unsuitable for forming hydrogen‐encapsulated superatoms. It therefore appears that the possibility for encapsulating hydrides in Ni‐centered superatomic cores is worthy of exploring, as well as their potential impact on catalytic properties. This is what we did in the work reported below. A one‐pot reduction method was employed to incorporate Ni into an Ag‐rich 8‐electron superatom, leading to the formation of [NiAg_20_{S_2_P(O*^i^* Pr)_2_}_12_] (**NiAg_20_**) and [NiHAg_19_{S_2_P(O*^i^* Pr)_2_}_12_] (**NiHAg_19_**). Their compositions and structural features were determined using SCXRD, ESI‐TOF‐MS, XPS, UV–vis, and multinuclear NMR spectroscopy. The presence of the hydride was further confirmed by NMR spectroscopy and supported by DFT calculations. For the first time, we found that the hydride in **NiHAg_19_** exhibits similar bonding features as in related Pd or Pt species. Furthermore, **NiAg_20_** exhibits oxygen evolution reaction (OER) activity, and hydride incorporation leads to a marginal improvement in the charge‐transfer kinetics during catalysis.

## 2 Results and Discussion

**Scheme 1:**
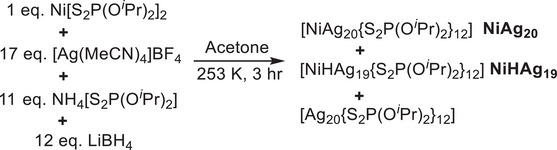
Synthesis of **NiAg_20_** and **NiHAg_19_** in acetone at 253 K.

[NiAg_20_{S_2_P(O*^i^* Pr)_2_}_12_] **NiAg_20_** and [NiHAg_19_{S_2_P(O*^i^* Pr)_2_}_12_] **NiHAg_19_** were synthesized via a direct reduction method. The organic‐soluble precursors, [Ag(MeCN)_4_]BF_4_ and [Ni{S_2_P(O*^i^* Pr)_2_}_2_], were reduced by LiBH_4,_ in the presence of dtp ligands ([S_2_P(O*^i^* Pr)_2_]^−^), with the reaction proceeding for a relatively short time of 3 h (**Scheme** **1**). **NiAg_20_** and **NiHAg_19_** were isolated in 19% and 13% yield by column chromatography using Al_2_O_3_ and a mixed mobile phase (dichloromethane and diethyl ether). A deuterated analogue, [NiDAg_19_{S_2_P(O*^i^* Pr)_2_}_12_] **NiDAg_19,_** was also obtained in 11% yield following a similar synthesis, by substituting LiBH_4_ with NaBD_4_ as the reducing reagent. The results could not be reproduced by using the anti‐galvanic exchange method.

**Figure 1:**
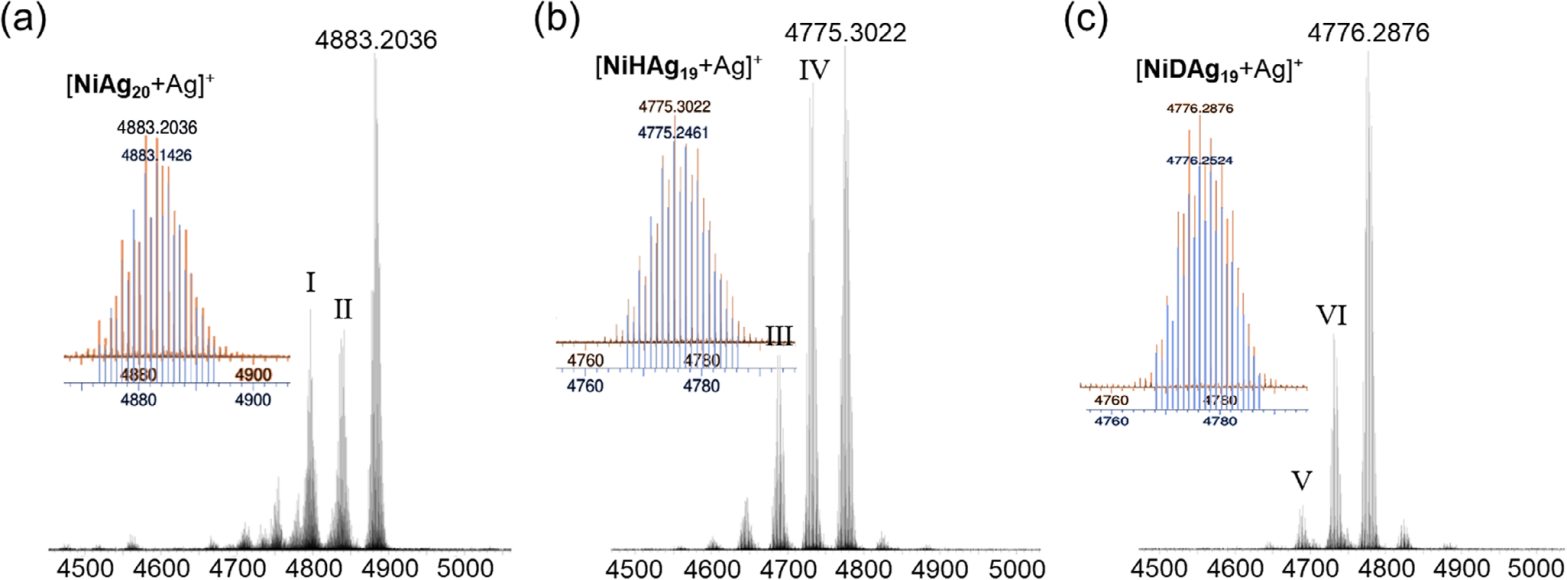
Experimental and theoretical isotopic distribution of positive‐ion ESI‐TOF‐MS spectra for a) [**NiAg_20_** + Ag]^+^, b) [**NiHAg_19_** + Ag]^+^, and for c) deuterated analog [**NiDAg_19_** + Ag]^+^.

The compositions of **NiAg_20_** and **NiHAg_19_** were confirmed by positive ESI‐MS spectroscopy. Silver adducts were observed, [**NiAg_20_** +Ag]^+^ at *m/z* 4883.2036 (calc. *m/z* 4883.1426) and [**NiHAg_19_** +Ag]^+^ at *m/z* 4775.3022 (calc. *m/z* 4775.2461), as shown in **Figure** **1**a and 1b. **NiDAg_19_** also showed a similar silver adduct fragment [**NiDAg_19_** +Ag]^+^ at *m/z* 4776.2876 (calc. *m/z* 4776.2524) in Figure 1c. The correlation between experimental and simulated isotopic distributions also shows good agreement. The remaining I‐VI peaks are assigned to sodium and solvate adducts (Figure S1, Supporting Information). Besides, XPS further verified the NC compositions, confirming the valence state of Ni and Ag for **NiAg_20_** and **NiHAg_19_** in Figure S2 (Supporting Information). The Ni 2p peaks of **NiAg_20_** were observed at 852.62 and 869.87 eV, corresponding to the Ni^0^ binding energies of 2p_3/2_ and 2p_1/2_, and similarly for **NiAg_19_** (852.72 and 870.27 eV).

**Figure 2:**
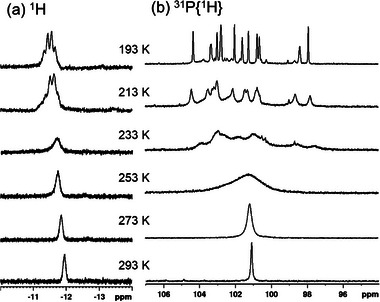
VT multinuclear NMR spectra of **NiHAg_19_** from 293 to 193 K in CDCl_3_ a) ^1^H NMR and b) ^31^P{^1^H} NMR.

The solution ^1^H NMR spectroscopy provided clear evidence for the inclusion of a hydride in **NiHAg_19_**, revealing a broad resonance centered at δ = −11.96 ppm at 293 K (**Figure** **2**a). Further analysis of the ^1^H NMR spectra confirmed a ligand:hydride ratio of 12:1 (Figure S3, Supporting Information). ^2^H NMR spectroscopy was performed on the deuterated analog, **NiDAg_19_**, to further verify the hydride magnetic environment. This yielded a single resonance at δ = −11.82 ppm (Figure S4, Supporting Information), correlating to the chemical shift observed in the ^1^H NMR and thus confirming the hydride environment. In contrast, no hydride resonance was observed in **NiAg_20_**, with ligand resonances observed at the expected positions and ratios (Figure S5, Supporting Information). Variable‐Temperature NMR (VT‐NMR) provided further crucial evidence regarding the hydride environment. Upon cooling to 193 K, the previously broad resonance forms a distinct quartet at δ = −11.44 ppm (Figure 2a) with coupling constants ^1^ *J*_1H‐107 Ag_ = 54.94 Hz and ^1^ *J*_1H‐109 Ag_ = 56.53 Hz. The coupling pattern suggests the hydride closely interacts with three chemically equivalent silver atoms. This observation is consistent with the hydride located within a NiAg_3_ tetrahedral cavity of the Ag_12_ icosahedron, a structural feature similar to those found in the related [MHAg_19_(L)_12_] (M = Pd, Pt; L = dtp, dsep).^[^ ^31^, ^33^, ^34^ ^]^ The dynamic behavior of the external dtp ligands was investigated using VT ^31^P{^1^H} NMR spectroscopy. At ambient temperature, **NiHAg_19_** and **NiAg_20_** display a single phosphorus peak at 101.09 ppm (Figure 2b), and 99.93 ppm (Figure S6, Supporting Information), respectively. However, upon cooling to 173 K, this single resonance of **NiHAg_19_** splits into multiple peaks ranging from 97.48 to 104.39 ppm (Figure 2b). This splitting is consistent with the solid‐state *C*_1_ symmetry of **NiHAg_19_**, where each ligand occupies a unique chemical environment at lower temperatures due to reduced lability of the passivating layer or conformational changes.

**Figure 3:**
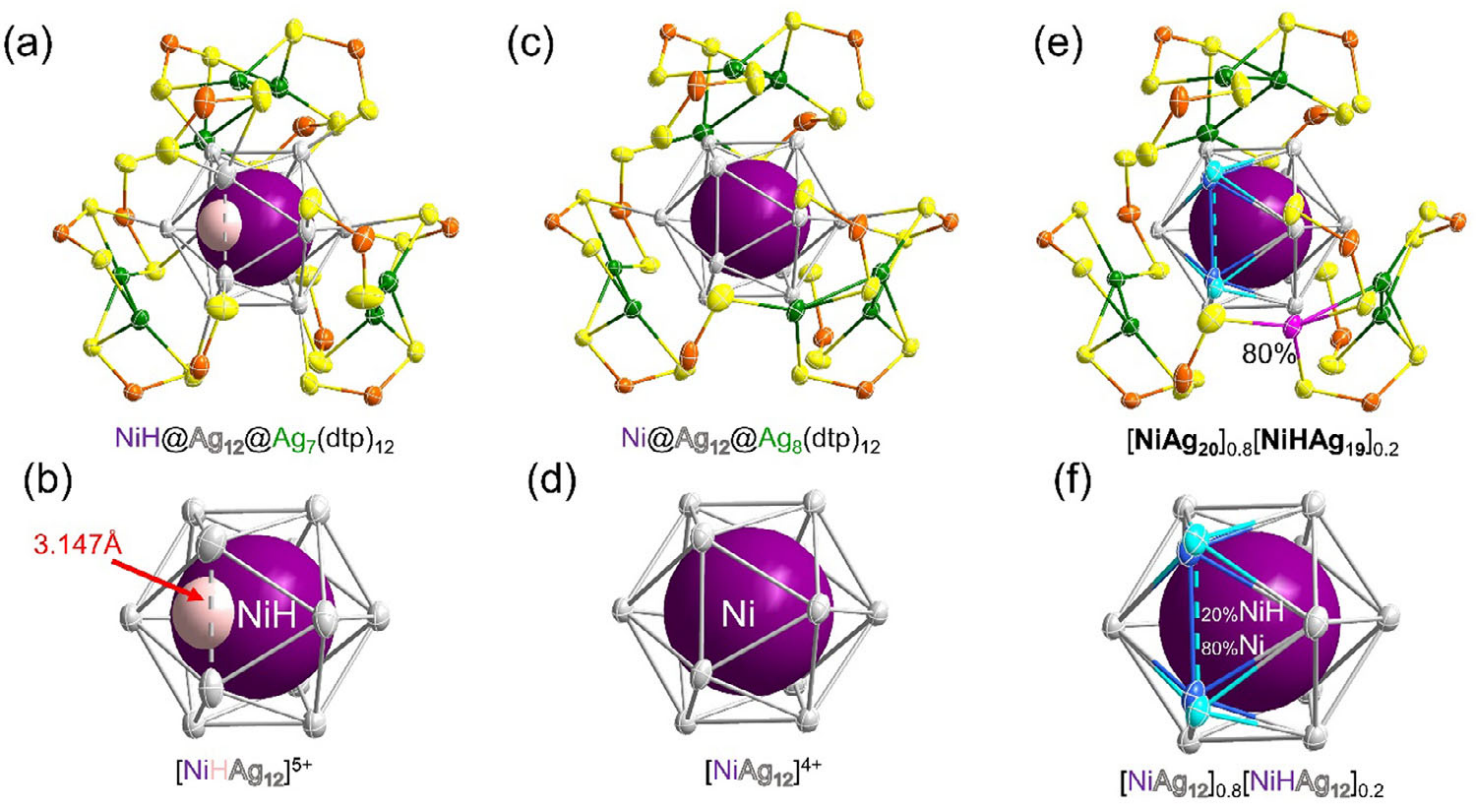
Total structure of a) **NiHAg_19_**_,_ c) **NiAg_20,_** and e) co‐crystal. Superatomic core of b) **NiHAg_19_**_,_ d) **NiAg_20_**_,_ and f) co‐crystal. (Color code: NiH/Ni = purple, Ag_ico_ = gray and blue, Ag_cap_ = green and pink, S = yellow, P = orange, H = pale pink).

Single crystals of **NiHAg_19_** and **NiAg_20_** can be obtained using hexane as the crystallization solution. Adding a more polar solvent such as methanol to this solution results in the spontaneous transformation of **NiHAg_19_** into **NiAg_20_**. This behavior has been confirmed by the observation of additional experiments, which will be discussed further down. Their crystal structures were determined by SCXRD. (Table S1, Supporting Information) Selected bond lengths are listed in **Table** **1**. The [(NiH)@Ag_12_]^5+^ icosahedral core of **NiHAg_19_** is slightly distorted, due to the presence of the encapsulated hydrogen within one of the twenty NiAg_3_ tetrahedra (**Figure** **3**b). This position was later confirmed by DFT calculations (see below). This distortion can be evaluated by its continuous symmetry measure (CSM)^[^ ^39^ ^]^ value, which is 0.09. The [Ag_7_(dtp)_12_]^5−^ passivating layer of **NiHAg_19_** is analogous to that of [Ag_20_(dtp)_12_] (**Ag_20_**),^[^ ^40^ ^]^ consisting of seven Ag capping (Ag_cap_) atoms capping triangular faces of the icosahedron, and linked to twelve dtp ligands (Figure 3a). The [Ni@Ag_12_]^4+^ icosahedral core of **NiAg_20_** is nearly regular (CSM = 0.06). It is face‐capped by eight Ag_cap_ atoms, giving rise to an overall *C*_2_ symmetry, which reduces to *C*_1_ upon coordination with the twelve dtp ligands (Figure 3c‐d; Figure S7, Supporting Information). In the hydride‐containing **NiHAg_19_**, Ni‐Ag_ico_ (Ag_ico_, icosahedral Ag) distances range from 2.666(1) to 2.907(2) Å, with an average of 2.745(1) Å, which is slightly shorter than those observed in PdHAg_19_(dtp)_12_ and PtHAg_19_(dtp)_12_.^[^ ^31^, ^33^ ^]^ The Ag_ico_–Ag_ico_ distances span from 2.813(1) to 3.147(1) Å, averaging 2.888(1) Å, representing a noticeable contraction relative to the Pd‐ and Pt‐doped analogues. These structural differences are reflected in the bond distance profiles shown in Figures S8 and S9 (Supporting Information). In **NiAg_20_**, an even more compact icosahedron is observed. The Ni–Ag_ico_ distances range from 2.646(2) to 2.819(2) Å, averaging 2.722(2) Å, while the Ag_ico_–Ag_ico_ distances range from 2.776(1) to 2.951(1) Å, with an average of 2.863(1) Å. To date, this represents the most compact icosahedral core among all reported eight‐electron superatoms stabilized by dtp ligands, with a compactness comparable to that of [NiAg_24_(SPhMe_2_)_18_]^2–^.^[^ ^16^ ^]^ The distinctions in bond lengths between **NiAg_20_** and its group 10 analogues are readily apparent in Figures S10 and S11 (Supporting Information).

**Table 1:** Selected Experimental Bond Distances (Å) **NiHAg_19_** and **NiAg_20_** and DFT‐computed data (NAO = natural atomic orbitals; WBI = Wiberg bond indices).

	NC	NiHAg_19_	NiAg_20_
	CSM	0.09	0.06
	Ni‐Ag_ico_	2.666(1)‐2.907(2) avg. 2.745(1)	2.646(2)‐2.819(2) avg. 2.722(2)
	Ag_ico_‐Ag_ico_	2.813(1)‐3.147(1) avg. 2.888(1)	2.776(1)‐2.951(1) avg. 2.863(1)
	Ag_ico_‐Ag_cap_	2.885(1)‐3.106(1) avg. 2.989(1)	2.848(1)‐3.096(1) avg. 2.977(1)

DFT			
NAO charges (avg.)	CSM	0.12	0.04
	Ni	−0.75	−0.91
	H	−0.49	−
	Ag_ico_	0.33	0.26
	Ag_cap_	0.70	0.70
Distances [WBI] (avg.)	Ni─H	1.582 [0.143]	−
	Ag‐H	1.972 [0.110]	−
	Ni‐Ag_ico_	2.833 [0.112]	2.798 [0.119]
	Ag_ico_‐Ag_ico_	2.959 [0.083]	2.942 [0.094]
	Ag_ico_‐Ag_cap_	3.115 [0.043]	3.098 [0.045]

When the crystallization is performed in methanol, **NiHAg_19_** and **NiAg_20_** co‐crystallize in a 1:4 ratio, i.e., [**NiHAg_19_**]_0.2_[**NiAg_20_**]_0.8_, giving rise to a disordered structure favored by their structural similarities. (Figures S12–S15, Supporting Information) Due to differences in the number of Ag_cap_ atoms and the degree of icosahedral distortion between **NiHAg_19_** and **NiAg_20_**, two reasonable structural models can be distinguished. In [**NiHAg_19_**]_0.2_[**NiAg_20_**]_0.8_, one Ag_cap_ atom exhibits a site occupancy of only 0.8, and the icosahedral core can be divided into two configurations: a regular icosahedron with 0.8 occupancy and a distorted icosahedron with 0.2 occupancy. (Figure 3e‐f; Figure S12, Supporting Information)

**Scheme 2:**
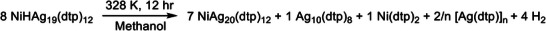
The self‐redox reaction of **NiHAg_19_** under heating for 12 h in methanol.

The co‐crystallization phenomenon drew our attention to the spontaneous transformation of **NiHAg_19_** into **NiAg_20_** in polar solvents, which prompted us to monitor this conversion process via solution NMR spectroscopy. The process can be accelerated by heat and tracked by ^31^P{^1^H} NMR spectroscopy. Figure S14 (Supporting Information) illustrates the conversion of **NiHAg_19_ (**δ = 101.1 ppm) to **NiAg_20_** (δ = 99.9 ppm), over a period of 12 h at 328 K in *d*_4_‐methanol. Additional peaks at 89.3, 104.3, and 107.6 ppm are observed, which are assigned to byproducts Ni(dtp)_2_, Ag_10_(dtp)_8_, and polymeric [Ag(dtp)]_n_, respectively. Besides, in‐situ generated H_2_ gas was detected in the ^1^H NMR spectrum at 4.5 ppm (Figure S15, Supporting Information). These findings suggest a complex redox transformation of **NiHAg_19_**. During this process, the encapsulated hydride and nickel(0) are oxidized to H_2_ gas and nickel(2+), respectively. Simultaneously, a partial reduction of silver(1+) to silver(0) occurs. (**Scheme** **2**).

**Figure 4:**
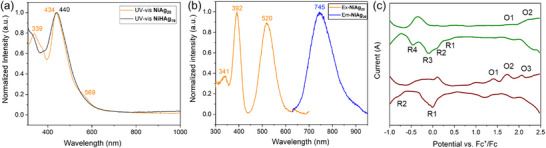
a) UV–vis spectra for **NiHAg_19_** and **NiAg_20_**. b) Excitation and Emission spectra for **NiAg_20_**. c) SWVs of **NiAg_20_** (green) and **NiHAg_19_** (brown) in CH_2_Cl_2_ containing 0.1 m Bu_4_NPF_6_ at 233 K.

The optical properties of **NiAg_20_** and **NiHAg_19_** were investigated using UV–vis and photoluminescence (PL) spectroscopy. The absorption spectra of the two Ni‐Ag NCs differ significantly. **NiHAg_19_** displays a single peak at 440 nm, while **NiAg_20_** shows peaks at 339 and 434 nm, along with a shoulder at 569 nm (**Figure** **4**a). In the PL spectrum recorded at 77 K, **NiAg_20_** exhibits an emission peak at 745 nm, which is comparable to the emission features of PdAg_20_(dtp)_12_ (**PdAg_20_**; 741 nm) and PtAg_20_(dtp)_12_ (**PtAg_20_**; 677 nm), indicating similar emissive behavior among the group 10‐doped NCs.^[^ ^30^, ^32^, ^36^ ^]^ The emission lifetime recorded for **NiAg_20_** is 110 µs (Figure S16, Supporting Information). It is significantly shorter compared to the radiation decay of **PdAg_20_** (235 µs).^[^ ^41^ ^]^ The stability of **NiHAg_19_** was confirmed by the time‐dependent absorption spectra, shown in Figure S17 (Supporting Information).

The electrochemical properties of **NiAg_20_** and **NiHAg_19_** were investigated in CH_2_Cl_2_ solution containing 0.1 m Bu_4_NPF_6_ at 233 K. In Figure 4c (green), the square wave voltammetry (SWV) of **NiAg_20_** exhibits four reduction processes and two oxidation processes (Figure 4c and **Table** **2**). Its electrochemical HOMO–LUMO gap (1.47 V) is much less than those of **PdAg_20,_^[^** ^41^ ^]^ and **PtAg_20_^[^** ^27^ ^]^ (Table 2). The SWV of **NiHAg_19_** shows two reduction processes and three oxidation peaks (Figure 4c and Table 2). The cyclic voltammetry (CV) of **NiAg_20_** and **NiHAg_19_** (Figure S18, Supporting Information) show similar current patterns to the SWV curves. The electrochemical HOMO‐LUMO gaps of **NiAg_20_** and **NiHAg_19_** are similar to that reported for [NiAg_24_(SPhMe_2_)_18_]^2–^,^[^ ^16^ ^]^ in line with their similar superatomic 8‐electron electronic structures.

**Table 2:** The redox potentials versus Fc^+^/Fc(v) and electrochemical HOMO‐LUMO gap *E*_g_ = *E*_pa_ – *E*_pc_ (all values in eV).

NC	*E*_pc_	*E*_pa_	*E*_g_
PdAg_20_	−1.86	−0.15, +0.09,+0.51, +0.90	1.71
PtAg_20_	−1.98	−0.06, +0.16, +0.48, +0.71	1.92
NiAg_20_	+0.35, +0.11, –0.09, –0.46	+1.82, +2.22	1.47
NiHAg_19_	−0.00, –0.77	+1.41, +1.73, +2.08	1.41

**Figure 5:**
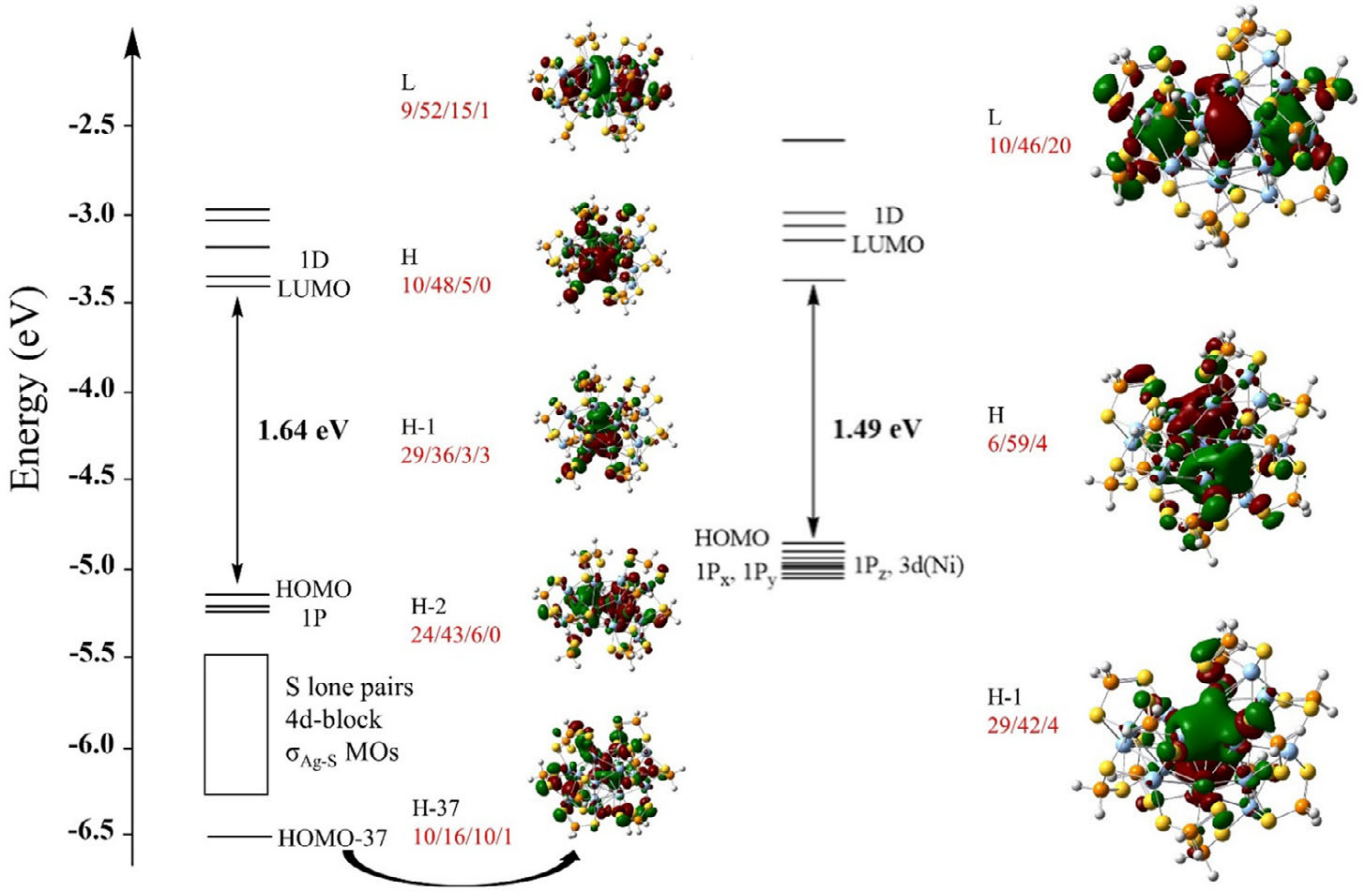
Kohn–Sham frontier orbital diagrams of **NiHAg_19_** and **NiAg_20_** (H = HOMO; L = LUMO). Values in red are the contribution (in %) of the core atoms or atom groups in the following order: Ni/Ag_ico_/Ag_cap_/H. (isovalue = 0.02).

Compounds **NiHAg_19_** and **NiAg_20_** were investigated by DFT calculations at the BP86/def2‐TZVP level (see Computational Details in the Supporting Information). To reduce computational cost, the dtp ligand was replaced by the simplified S_2_PH_2_ model, a strategy previously validated in many similar systems.^[^ ^11^, ^12^, ^28^, ^29^, ^30^, ^31^, ^33^, ^34^, ^35^, ^37^, ^40^ ^]^ Selected computed data are given in Table 1. The computed metrical data are consistent with their SCXRD homologues and, as expected, the encapsulated hydrogen in **NiHAg_19_** occupies a distorted (enlarged) NiAg_3_ cavity. In both **NiHAg_19_** and **NiAg_20_**, the NBO charges of the peripheral Ag_cap_ atoms (+0.7) are consistent with a +1 oxidation state, as expected from their trigonal planar coordination environment. In contrast, the significantly lower charges on the Ag_ico_ atoms (+0.3) suggest mixed‐valent character. The NBO charge of the hydride in **NiHAg_19_** is comparable to values reported for hydrides embedded in other superatomic cores.^[^ ^24^, ^31^, ^33^, ^34^, ^35^, ^37^ ^]^ As a whole, the computed data are consistent with the view of **NiHAg_19_** and **NiAg_20_** as made of [NiHAg_12_]^5+^ and [NiAg_12_]^4+^ 8‐electron cores stabilized by [Ag_7_(dtp)_12_]^5−^ and [Ag_8_(dtp)_12_]^4−^ passivating layers, respectively. Their Kohn–Sham orbital diagrams (**Figure** **5**) are in line with their closed‐shell 1S^2^ 1P^6^ 1D^0^ superatomic configuration. Thus, although the icosahedral core in **NiHAg_19_** is distorted, the cluster nonetheless conforms to a closed‐shell 8‐electron superatomic configuration. As in PtHAg_19_(dtp)_12_^[^ ^31^ ^]^ and PdHAg_19_(dtp)_12_^[^ ^33^ ^]^ the hydride contributes modestly to the superatomic orbitals (Figure 5), indicating that, although the encapsulated H atom is part of the superatomic 8‐electron manifold, the 1s(H) orbital is not significantly involved in the construction of the superatomic orbitals.^[^ ^24^ ^]^ Rather, it interacts strongly with one of the 3d(Ni) orbitals, making a fairly localized Ni─H σ‐bond, as illustrated by the corresponding bonding combination (HOMO–37) shown in Figure S19 (Supporting Information).

At this stage of the discussion, it is worth noting that, although many nickel hydride complexes have been reported,^[^ ^42^ ^]^ as far as we know, the oxidation state of Ni in all these species is larger than zero (most often +II), if one puts aside the solid state compound Mg_2_NiH_4_, which contains tetrahedral [NiH_4_]^4−^ anions.^[^ ^43^ ^]^ With respect to nickel hydride clusters, there is only a very small number of systems in which the Ni oxidation state is very close to zero.^[^ ^44^, ^45^, ^46^ ^]^ Thus, **NiHAg_19_** is a very rare example of a structurally characterized Ni(0) hydride compound.

The TD‐DFT UV–vis spectra of **NiHAg_19_** and **NiAg_20_**, simulated at the CAM‐B3LYP/Def2‐TZVP level (see Computational Details in the Supporting Information) are shown in Figure S20 (Supporting Information). They exhibit slight deviations from the experimental results, which may be attributed to the fact that not all possible isomers were considered in the calculations. The lowest‐energy absorption bands in **NiHAg_19_** and **NiAg_20_** are primarily attributed to electronic transitions of 1P→1D nature, mixed with some 3d(Ni)→1D character in the case of **NiAg_20_**.

**Figure 6:**
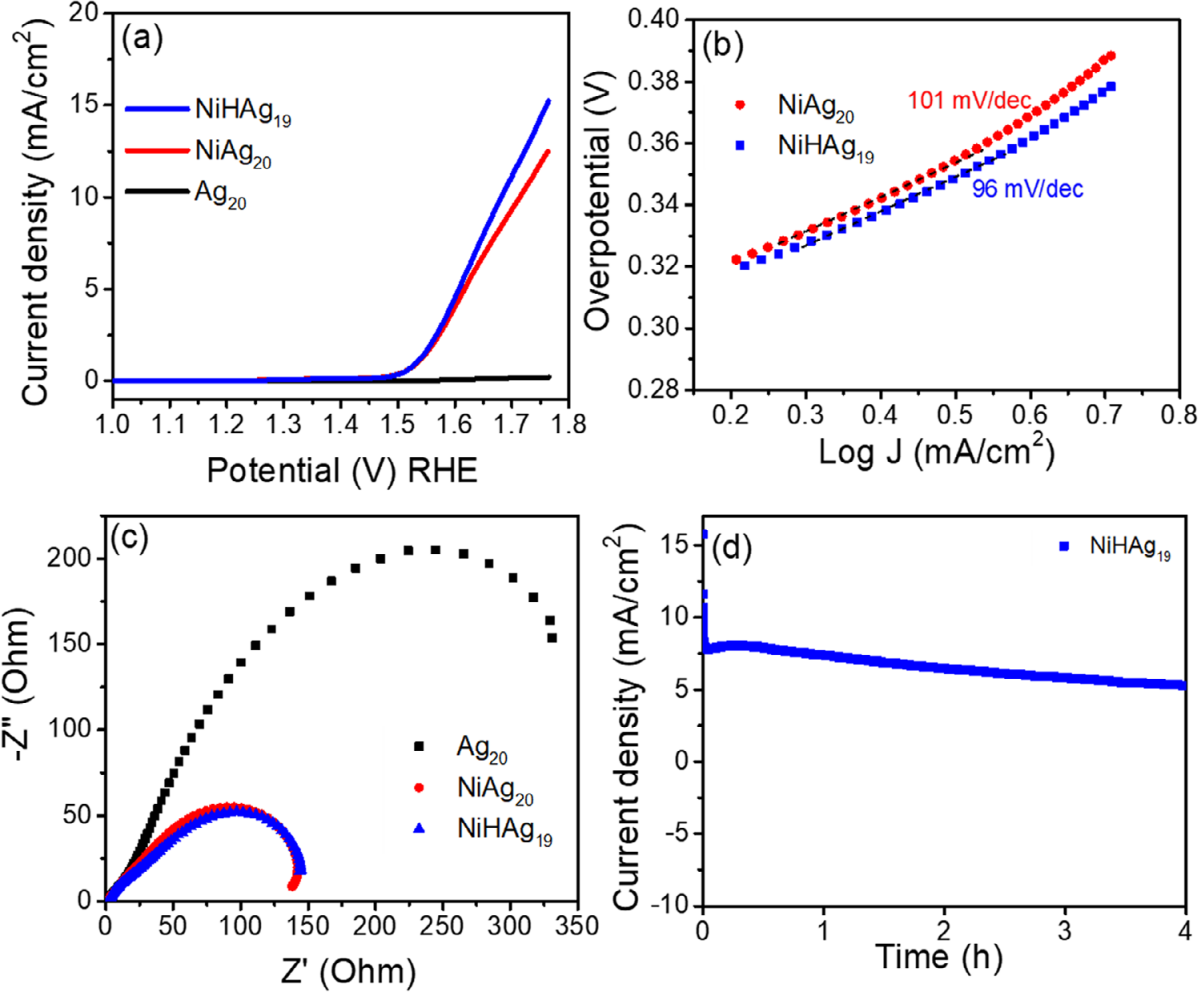
a) Linear sweep voltammetry curves recorded at a scan rate of 5 mV s^−1^ for **Ag_20_**, **NiAg_20,_** and **NiHAg_19_**; b)The Tafel slopes of **NiAg_20_** and **NiHAg_19_**; c) EIS of **Ag_20_**, **NiAg_20_** and **NiHAg_19_**; d) chronoamperometry study of **NiHAg_19_** showing the stability of the catalyst for 4 h.

The oxygen evolution reaction (OER) performance of **NiAg_20_** and **NiHAg_19_** NCs was evaluated in 1 m KOH and compared with their monometallic **Ag_20_** relative, as well as the Ni(dtp)_2_ complex. The cyclic voltammetry responses of the compounds showed an anodic activation, and the current densities were stabilized with CV cycles, as shown in Figure S21 (Supporting Information). Linear sweep voltammetry (LSV) curves presented in **Figure** **6**a show that pristine **Ag_20_** displays poor anodic current even at higher potentials, affirming the inertness of silver toward the oxygen evolution reaction.^[^ ^47^ ^]^ On the contrary, **NiAg_20_**, and **NiHAg_19_** show a notable improvement in the OER. The LSV curves for the Ni‐doped NCs show a more gradual increase in current compared to Ni(dtp)_2_, suggesting that while Ni doping enhances activity over bare Ag_20_, the overall charge transfer kinetics remains limited by the spatial inaccessibility of the Ni atom embedded within the metallic core.^[^ ^48^ ^]^ To further study the electrocatalytic efficiency of the compounds, the overpotential required to achieve a current density of 10 mA cm^−2^ was evaluated and compared to Ni(dtp)_2_. The latter is reaching this current density at an impressively low overpotential of 350 mV (Figure S22a, Supporting Information), underscoring its rapid kinetic response during OER. In comparison, **NiAg_20_** and **NiHAg_19_** require overpotentials of 490 and 450 mV, respectively, reflecting their comparatively sluggish behavior. Electrocatalytic kinetics were further studied by evaluating Tafel plots (Figure 6b). Ni(dtp)_2_ exhibits a Tafel slope of 58 mV dec^−1^ (Figure S22b, Supporting Information), indicative of fast reaction kinetics. In comparison, **NiHAg_19_** and **NiHAg_20_** show Tafel slopes of 96 and 101 mV dec^−1^, respectively, both pointing toward slower charge‐transfer kinetics. Electrochemical impedance spectroscopy (EIS) was also performed to gain deeper insights into the interfacial charge transfer characteristics of the compounds. EIS offers a clear understanding of how efficiently charges move across the catalyst‐electrolyte interface. As shown in Figure 6c, the Nyquist plots showed clear differences. **Ag_20_** exhibits a large semicircular arc, indicative of high interfacial resistance. Interestingly, the Ni‐doped NCs present intermediate arc sizes, suggesting that incorporation of Ni helps electrons to move more easily across the interface. So, Ni is playing a beneficial role in enhancing conductivity or lowering charge transfer resistance. This is probably due to the significantly higher Ni hydration enthalpy, as compared to that of Ag, though both metals have similar electronegativity. This leads to the effective adsorption of OH^−^ ions at the catalyst's active sites.^[^ ^49^ ^]^ The durability of **NiHAg_19_** NCs for the OER was also evaluated by chronoamperometric (CA) studies. An enhanced durability of **NiHAg_19_** was recorded for 4 h with a minimum loss of initial current density (Figure 6d). Under similar experimental conditions, PdHAg_19_(dtp)_12_, and PtHAg_19_(dtp)_12_, showed lower OER activity than **NiHAg_19_**, further demonstrating the superior redox adaptability of the nickel center for OER.

The observed difference in catalytic activity between Ni(dtp)_2_ and the Ni‐doped NCs can be attributed to the distinct oxidation states and coordination environments of the Ni centers. In Ni(dtp)_2_, nickel exists in Ni^2+^ oxidation state, stabilized by strong coordination with dithiophosphate ligands. This configuration facilitates effective redox cycling during the catalytic process, allowing Ni to actively participate in charge transfer and reaction steps. The LSV of Ni(dtp)_2_ shows a strong peak near 1.34 V w.r.t RHE, corresponding to the oxidation of the Ni^2+^ to Ni^3+,^ forming the NiOOH, which is an active species for OER process.^[^ ^50^, ^51^, ^52^ ^]^ A recent study by Ahmed et al. reported the OER activity of the 8‐electron NC [NiAg_28_(BDT)_12_]^4−^ (BTD = 1,3‐benzene dithiol).^[^ ^50^ ^]^ This cluster shows enhanced activity compared to its monometallic [Ag_29_(BDT)_12_]^3−^ relative. Despite the lack of experimental structural information, the authors observed a pronounced hump at 1.4 V versus RHE, corresponding to the oxidation of Ni^2+^ to Ni^3+^, leading to the formation of nickel oxyhydroxide (NiOOH) during the oxidative sweep. This transformation is proposed to contribute to the improved activity and the lower overpotential of 417 mV required to reach a current density of 10 mA cm^−2^. In our Ni‐doped NCs, the Ni atom remains buried at the center of the metallic core, where its oxidation state is Ni^0^. While this electronic delocalization may enhance overall conductivity, it can limit the redox flexibility of Ni, reducing its direct involvement in the catalytic activity. However, it can influence electronically the surrounding silver atoms, participating indirectly in the redox processes. As a result, although the catalytic activity is improved compared to **Ag_20_**, it is still limited by the geometric inaccessibility of the Ni center and the lack of direct redox involvement. Thus, the higher catalytic activity observed for Ni(dtp)_2_ can be linked to the accessible and redox‐active Ni^2^⁺ center, while in our Ni‐doped NCs, the activity is influenced more by the overall NC architecture and electronic synergy, rather than by Ni‐centered redox processes alone.

## 3 Conclusion

While very few examples of structurally characterized Ni‐doped noble metal NCs are known to date, two novel species, **NiAg_20_** and **NiHAg_19_**, synthesized via a direct reduction method, are reported. Their atomically precise compositions and superatomic 8‐electron configurations were confirmed by SCXRD, ESI‐MS, NMR and supported by DFT calculations. In both cases the Ni atom occupies the cluster center and **NiHAg_19_** is the first example ever reported of an Ni‐bonded hydride encapsulated within an NC superatomic core, and a rare molecular species with Ni(0)─H bonding. VT‐NMR spectroscopy provided crucial evidence for the hydride dynamic environment and interaction with the silver framework. Investigations of the alkaline OER electrocatalytic performance of **NiAg_20_** and **NiHAg_19_** reveal that the incorporation of Ni into Ag‐rich NCs significantly enhances the catalyst activity, as compared to their monometallic silver counterpart or their Pd and Pt relatives. These findings open new avenues for designing cost‐effective, first‐row transition metal‐doped NCs with tailored properties for various catalytic applications.

## Conflict of Interest

The authors declare no conflict of interest.

## Supporting information



The authors have cited additional references within the Supporting Information. ^[53‐66]^ Deposition numbers 2477724 (for NiHAg_19_), 2477725 (for NiAg_20_) and 2477726 (for [NiHAg_19_]_0.2_[NiAg_20_]_0.8_) contain the supplementary crystallographic data for this paper. These data are provided free of charge by the joint Cambridge Crystallographic Data Centre and Fachinformationszentrum Karlsruhe Access Structures service.

## Data Availability

The data that support the findings of this study are available in the supplementary material of this article.
