# Supplementary material for: A Ni‐Bonded Hydride in Superatomic Silver Nanoclusters: Probing Synergistic Heteroatomic Core Effects on Oxygen Evolution Reactivity
Source: Small. 2025 Nov 11;21(51):e12495. doi: 10.1002/smll.202512495 (PMC12723327; doi:10.1002/smll.202512495)
Supplement: Supplementary file 1 — The authors have cited additional references within the Supporting Information. [53‐66] Deposition numbers 2477724 (for NiHAg19), 2477725 (for NiAg20) and 2477726 (for [NiHAg19]0.2[NiAg20]0.8) contain the supplementary crystallographic data for this paper. These data are provided free of charge by the joint Cambridge Crystallographic Data Centre and Fachinformationszentrum Karlsruhe Access Structures service. [file SMLL-21-e12495-s001.docx]

**§Supporting Information**

**A Ni-Bonded Hydride in Superatomic Silver Nanoclusters: Probing Synergistic Heteroatomic Core Effects on Oxygen Evolution Reactivity**

Yu-Rong Ni,^[a]§^ Tzu-Hao Chiu,^[a]§^ Rugma T P,^[a]^ Michael N. Pillay,^[a]^ Samia Kahlal,^[b]^ Jean-Yves Saillard*,^[b]^ and C. W. Liu*^[a]^

1. Department of Chemistry, National Dong Hwa University, Hualien 97401, Taiwan (Republic of China). E-mail: [chenwei@gms.ndhu.edu.tw](mailto:chenwei@gms.ndhu.edu.tw)
2. Univ Rennes, CNRS, ISCR-UMR 6226, F-35000 Rennes, France. Email: jean-yves.saillard@univ-rennes.fr

* Correspondence: Email, [chenwei@gms.ndhu.edu.tw](mailto:chenwei@gms.ndhu.edu.tw)

§ equal contribution

**Experimental Section**

1. **Chemicals and Characterizations**

Commercially acquired chemicals were all utilized precisely as supplied, including Lithium borohydride (LiBH_4_, 95%) and Sodium borodeuteride (NaBD_4_, 98% ). The starting materials, [Ag(MeCN)_4_]BF_4_^53^ and NH_4_[S_2_P(O*^i^*Pr)_2_]^54^ (dtp, dithiophosphate), and Ni(dtp)_2_^55^ were prepared using the procedure described in the literature. The organic solvents were purified using standard procedures. All manipulations were performed using normal Schlenck procedures in a N_2_ atmosphere. VT-multinuclear NMR spectra were recorded on a Bruker Advance III HD-500 MHz spectroscopy, operating at 500 MHz for ^1^H, 76.77 MHz for ^2^H, and 202.46 MHz for ^31^P{^1^H}. The chemical shift (δ) and coupling constants (J) are reported in ppm and Hz, respectively. ESI-TOF-MS spectra were recorded on a Fison Quattro Bio-Q (Fisons Instruments, VG Biotech, UK). The XPS spectra were recorded by using a PHI 5000 VersaProbe-Scanning ESCA Microprobe on an X-ray Photoelectron Spectrometer. The optical absorption spectra were recorded using an Agilent Cary-60 spectrometer. Emission, excitation, and lifetime decay were recorded on a HORIBA FluoroMax plus spectrometer. Shimadzu Nexis GC-2030 was used to analyze the gas products. The electrocatalysis was recorded on an Admiral Squidstat Plus potentiostat.

1. **Synthesis of Ni-Ag NCs**

0.26 g of the dithiophosphate ligand (dtp, [NH_4_][S_2_P(O*^i^*Pr)_2_], 1.12 mmol) and 0.05 g of Ni(dtp)_2_ (0.10 mmol) were dissolved in 30 mL of acetone in a reaction flask at 253 K. After 20 minutes, 0.66 g of [Ag(MeCN)_4_]BF_4_ (1.71 mmol) was added, and the color quickly changed to colorless. Subsequently, 0.6 ml of LiBH_4_ was added and reacted for 3 hours. The solvent was eliminated under a vacuum. The dichloromethane (DCM) and DI water were used to extract the residue. The organic layer was purified by column chromatography with Al_2_O_3_ and a pure DCM mobile phase. The clusters [NiAg_20_{S_2_P(O*^i^*Pr)_2_}_12_] **NiAg_20_** and [NiHAg_19_{S_2_P(O*^i^*Pr)_2_}_12_] **NiHAg_19_** were obtained by using mixed solvent DCM: ether (8:1) and (6:4) elution in 19% and 13% yield. (yield based on Ni).

**NiAg_20_**: ^1^H NMR (500 MHz, CDCl_3_, δ, ppm, 298 K): 4.85(septet, CH, 24H), 1.38 (d, CH_2_, 144H). ^31^P{^1^H} NMR (202.46 MHz, CDCl_3_, δ, ppm, 298 K): 99.93. ESI-MS (*m/z*): exp. 4883.2036 (calc. 4883.1426 for [**NiAg_20_** + Ag]^+^). UV-Vis [λ_max_ in nm, (ε in M^-1^cm^-1^)]: 339(35033), 434(47821), 569(11973). XPS (Calc.: Ni%1.75, Ag%35.09, S%42.11, P%21.05): Ni%1.84, Ag%24.01, S%42.32, P%31.83, and (binding energy, eV): Ag 3d_5/2_, 368.24; Ag 3d_3/2_, 374.23; Ni 2p_3/2_, 852.62 (2p_3/2,_ sat. 858.92); Ni 3d_1/2_, 869.87 (2p_1/2,_ sat. 874.77).

**NiHAg_19_**:^1^ H NMR (500 MHz, CDCl_3_, δ, ppm, 293 K): 4.93 (septet, CH, 24H), 1.35 (d, CH_2_, 144H), -11.95 (m, NiH, 1H). ^31^P{^1^H} NMR (202.46 MHz, CDCl_3_, δ, ppm, 293 K): 101.09. VT ^1^H NMR (500 MHz, CDCl_3_, δ, ppm, 173 K): -11.44 (^1^*J*_1H-107Ag_ = 54.94 Hz, ^1^*J*_1H-109Ag_ = 56.53 Hz). ESI-MS (*m/z*): exp. 4775.3022 (calc. 4775.2461 for [**NiHAg_19_** + Ag]^+^). UV-Vis [λ_max_ in nm, (ε in M^-1^cm^-1^)]: 445 (46840).

XPS (Calc.: Ni%1.79, Ag%33.93, S%42.86, P%21.43): Ni%1.03, Ag%25.26, S%42.71, P%31.00, and (binding energy, eV): Ag 3d_5/2_, 368.37; Ag 3d_3/2_, 374.37; Ni 2p_3/2_, 857.72 (2p_3/2,_ sat. 858.42); Ni 3d_1/2_, 870.27 (2p_1/2,_ sat. 875.27).

**NiDAg_19_**: The LiBH_4_ was substituted by NaBD_4_, other conditions were kept constant, [NiDAg_19_{S_2_P(O*^i^*Pr)_2_}_12_] in yield of 11%. ^2^H NMR (76.77 MHz, CHCl_3_, δ, ppm): -11.82. ESI-MS (*m/z*): exp. 4776.2876 (calc. 4776.2524 for [**NiDAg_19_**]^+^).

1. **SCXRD**

Single crystals were coated with paratone oil and mounted on the tip of a loop. Single-crystal X-ray diffraction (SCXRD) data were measured using *ω* scans of 0.5° per frame for 0.3/1.1 s using an XraLAB Synergy-S diffractometer with Cu Kα radiation (λ = 1.54184 Å). The diffraction pattern was indexed, and the total number of runs and images was based on the strategy calculation from the program CrysAlisPro (Rigaku, V1.171.44.113a, 2025). Single source at home/near, HyPix-Arc 100 diffractometer. The crystal was kept at a steady T = 100.00(10) K during data collection. Data reduction, scaling, and absorption corrections were performed using CrysAlisPro (Rigaku, V1.171.44.113a, 2025). A Gaussian absorption correction was performed using CrysAlisPro 1.171.44.113a (Rigaku Oxford Diffraction, 2025). Numerical absorption correction based on Gaussian integration over a multifaceted crystal model. Empirical absorption correction using spherical harmonics, implemented in the SCALE3 ABSPACK scaling algorithm. The structure was solved with Olex2.Solve 1.5 ^56^ structure solution program using the Charge Flipping solution method and by using Olex2 ^57^ as the graphical interface. The model was refined with a version of Olex2. refine 1.5 ^56^ using Gauss-Newton minimization.

1. **Electrocatalytic measurements**

All aqueous electrochemical measurements were performed in Argon-purged 1 M KOH prepared with DI water. The catalyst inks were prepared by dissolving 3 mg of the complex and 5 mg of activated carbon in 950 μL of methanol and 50 μL of Nafion solution and then drop casting 15 μL of the mixture onto the carbon paper electrode (1 cm^2^). Catalyst-immobilized carbon paper served as the working electrode. An Ag/AgCl electrode served as a reference electrode, and a Pt plate as a counter electrode. All electrochemical data were collected on a Squidstat Plus potentiostat (Admiral Instrument).

1. **Computational Details**

Geometry optimizations were carried out within the formalism of the density functional theory (DFT) with the Gaussian 16 package,^58^ using the BP86 functional^59-60^ and the Def2-TZVP basis set from EMSL Basis Set Exchange Library.^61-62^ All the optimized geometries were characterized as true minima or transition states by vibrational analysis. The compositions of the molecular orbitals were calculated using the AOMix program.^63^ The natural atomic orbital (NAO) charges and Wiberg bond indices were computed with the NBO 6.0 program.^64^ The UV-visible transitions were calculated on the above-mentioned optimized geometries by means of time-dependent DFT (TD-DFT) calculations, with the CAM-B3LYP functional^65^ and the Def2-TZVP basis set. The UV–visible spectra were simulated from the computed TD-DFT transition energies and their oscillator strengths by using the SWizard program,^66^ each transition being associated with a Gaussian function of half-height width equal to 2000 cm^-1^.


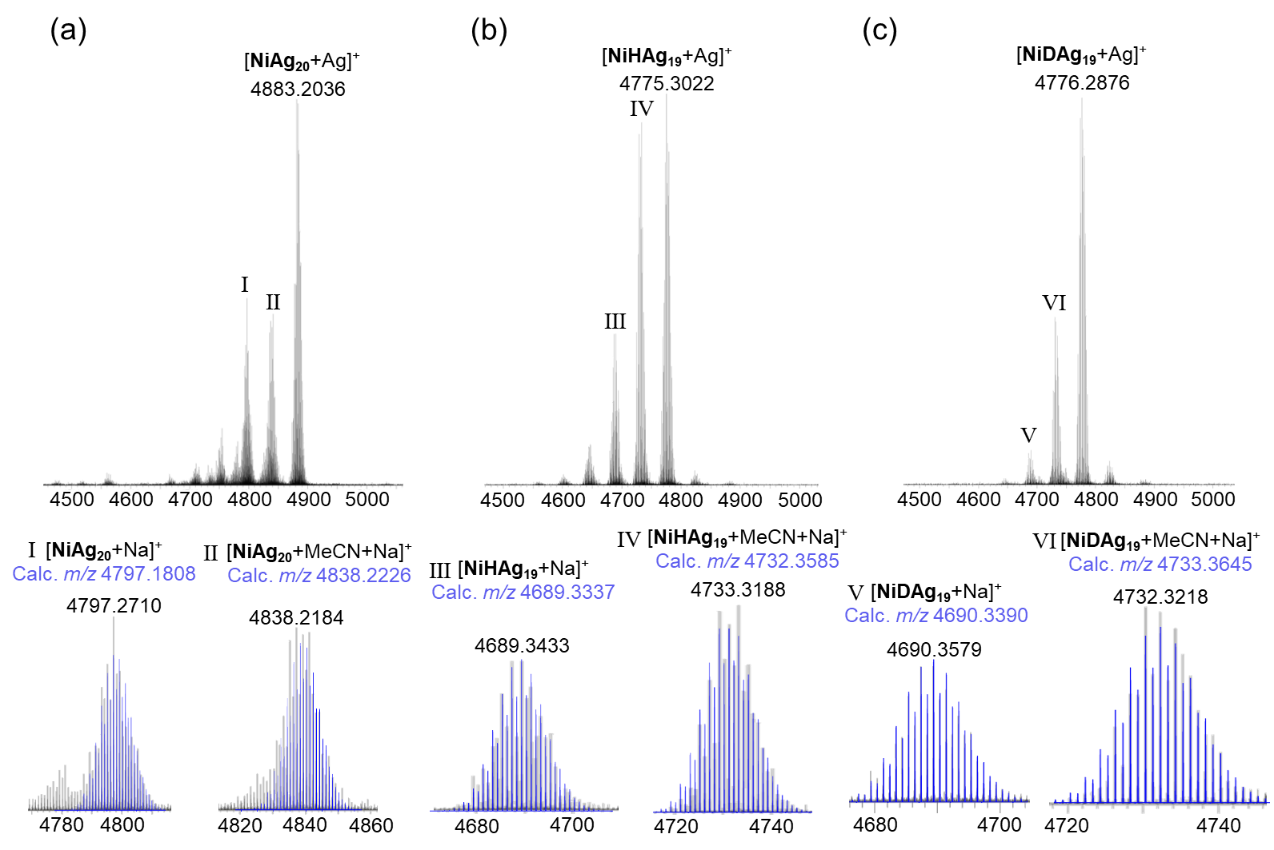


**Figure S1.** The positive-ion ESI-TOF-MS spectra for the remaining peaks Ⅰ-Ⅵ. The experimental (black) and simulated (blue) isotopic distributions are consistent.


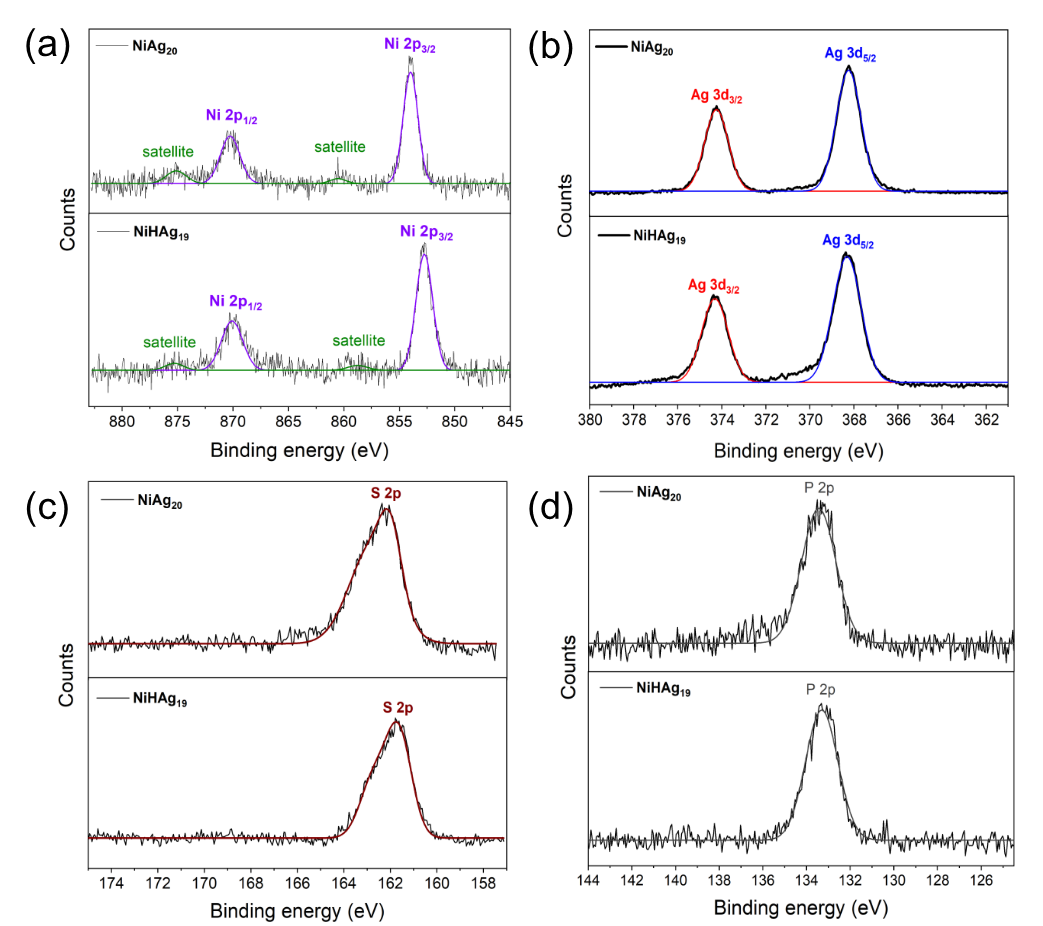


**Figure S2.** The XPS analysis of **NiAg_20_** and **NiHAg_19_** (a) Ni 2p XPS spectra, (b) Ag 3d XPS spectra. [S_2_P(O*^i^*Pr)_2_]^-^ ligands (S 2p, P 2p) XPS spectra of **NiAg_20_** and **NiHAg_19_** for (c) and (d).


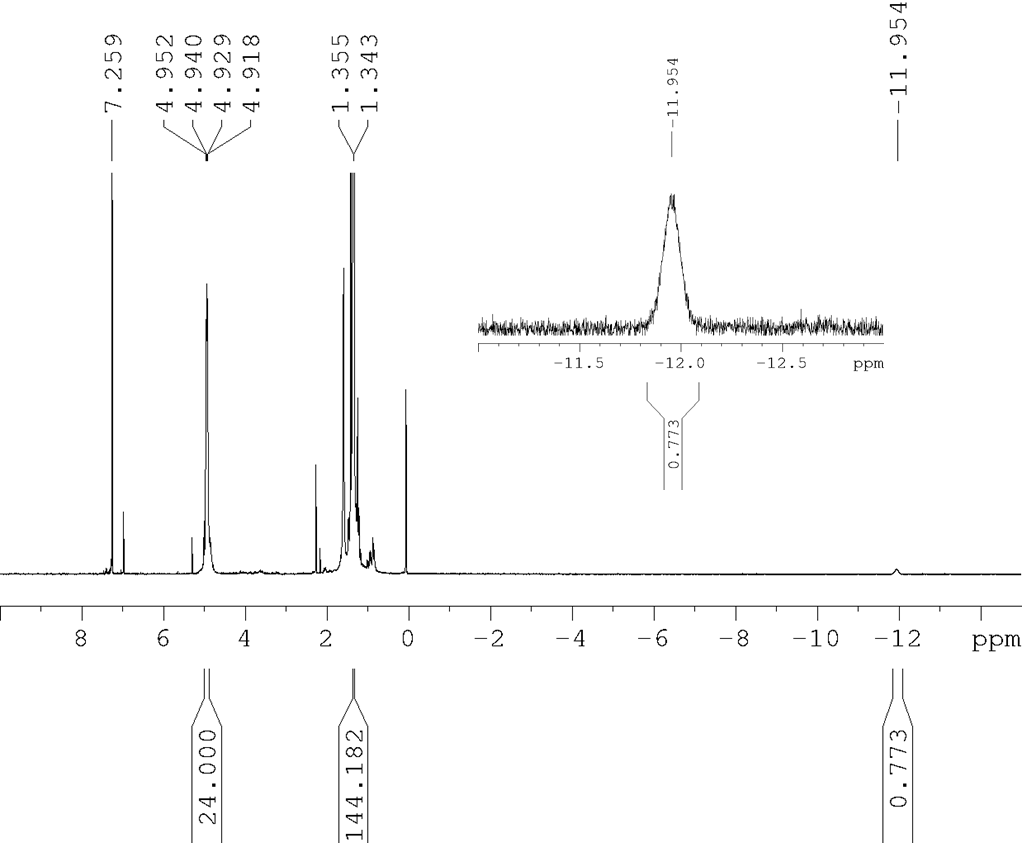


**Figure S3.** ^1^H NMR spectrum (500 MHz, CDCl_3_) of **NiHAg_19._**


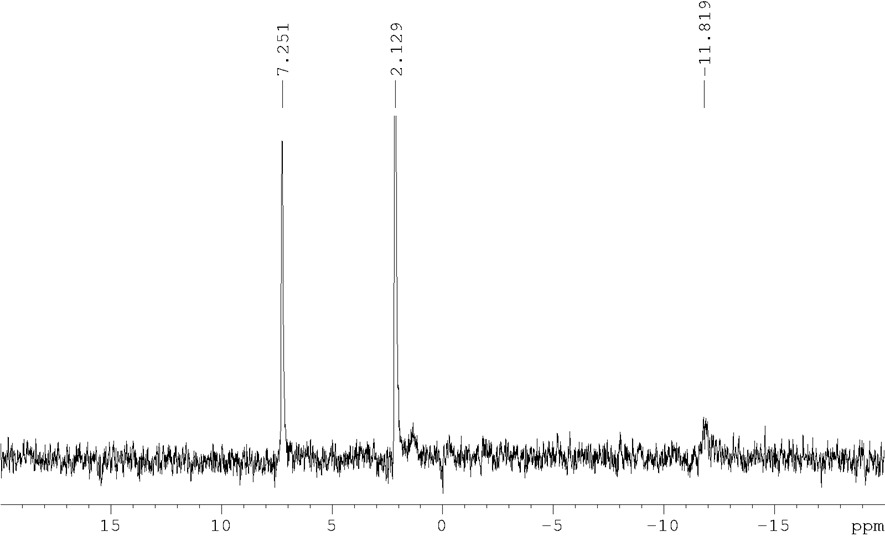


**Figure S4.** ^2^H NMR spectrum (76.77 MHz) of **NiDAg_19_** in CHCl_3_, 2.12 ppm is the acetone solvent peak in the spectrum.


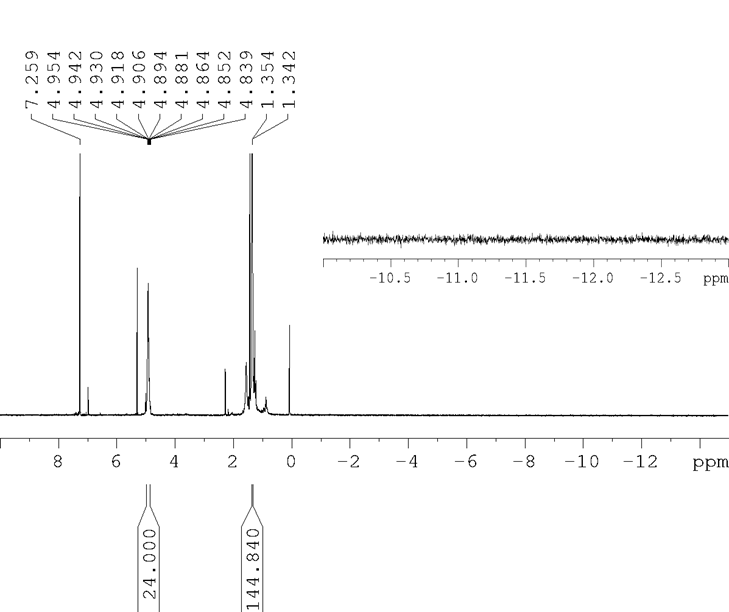


**Figure S5.** ^1^H NMR spectrum (500 MHz, CDCl_3_) of **NiAg_20._**


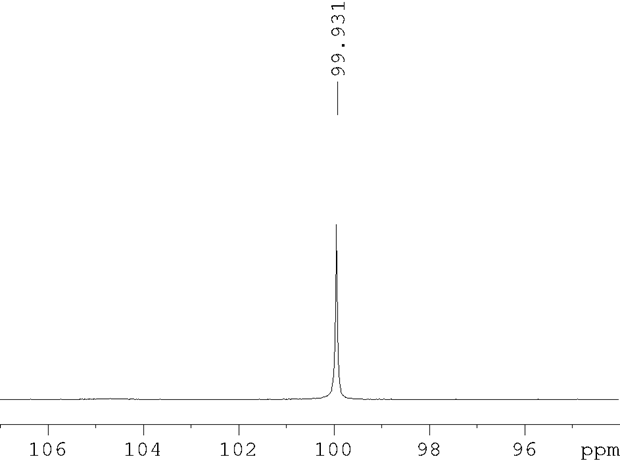


**Figure S6.** ^31^P NMR spectrum (202.46 MHz, CDCl_3_) of **NiAg_20._**


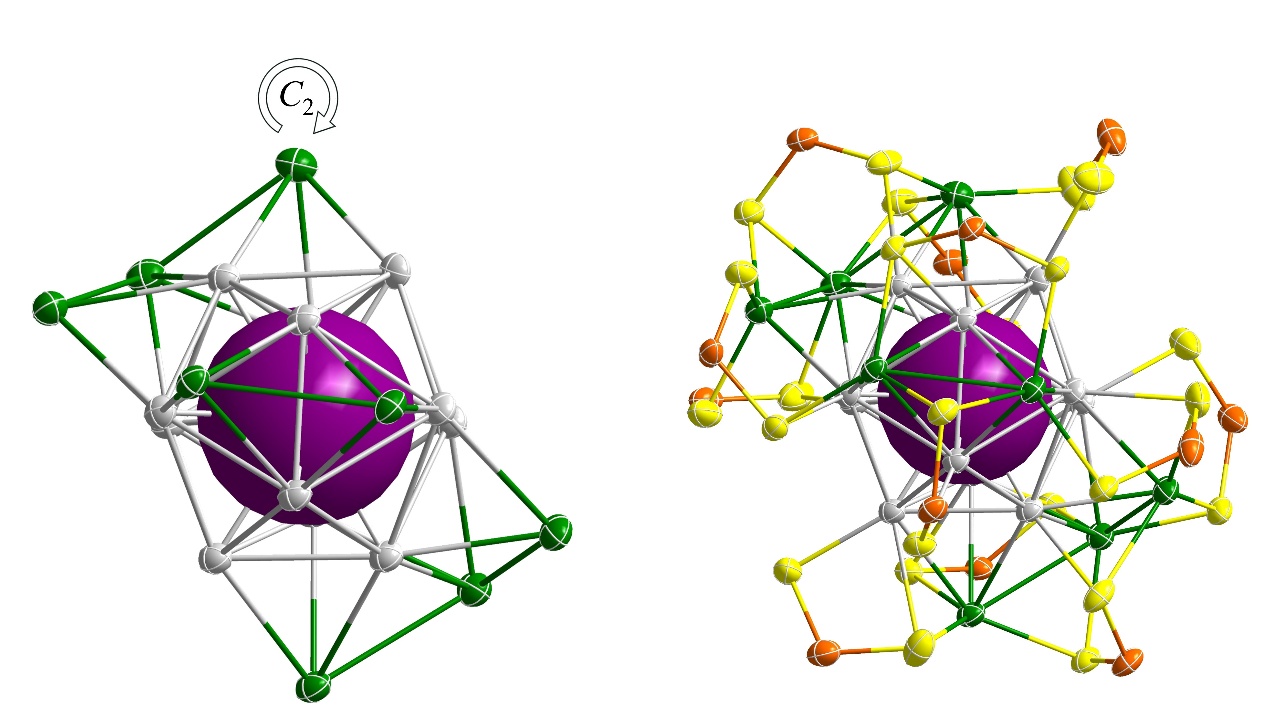


**Figure S7.** Metal framework (left) and total structure (right) of **NiAg_20_** (color code: purple: Ni; gray: Ag_ico_; green: Ag_cap_; yellow: S; orange: P)


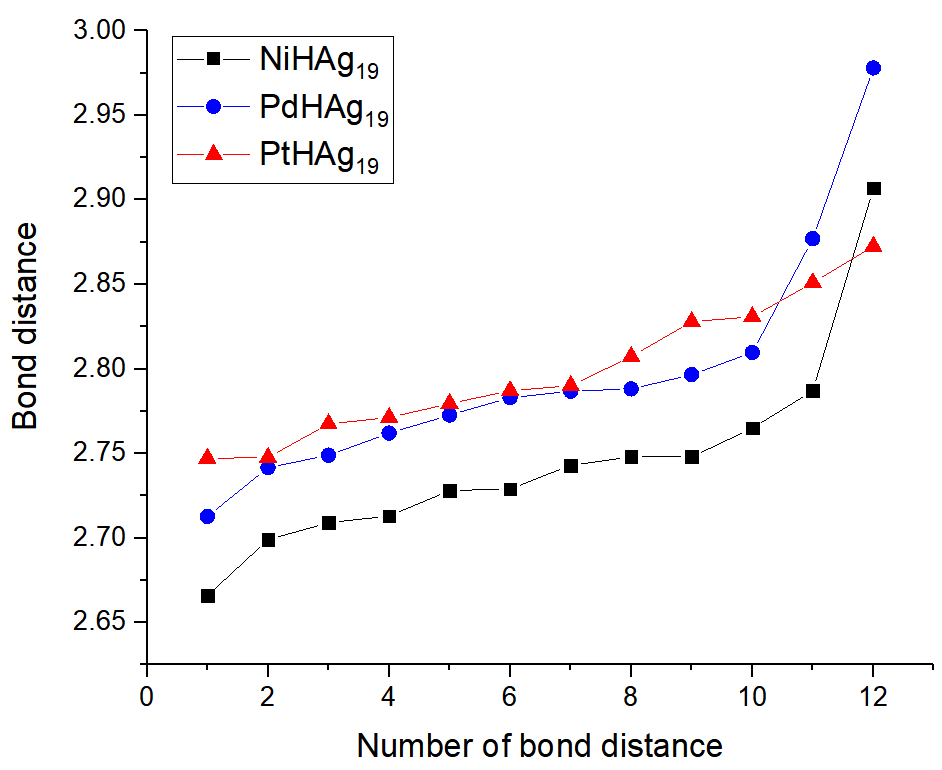


**Figure S8.** The distribution of Ni-Ag_ico_ distances in **NiHAg_19_** and MHAg_19_(dtp)_12_ (M = Pd, Pt).


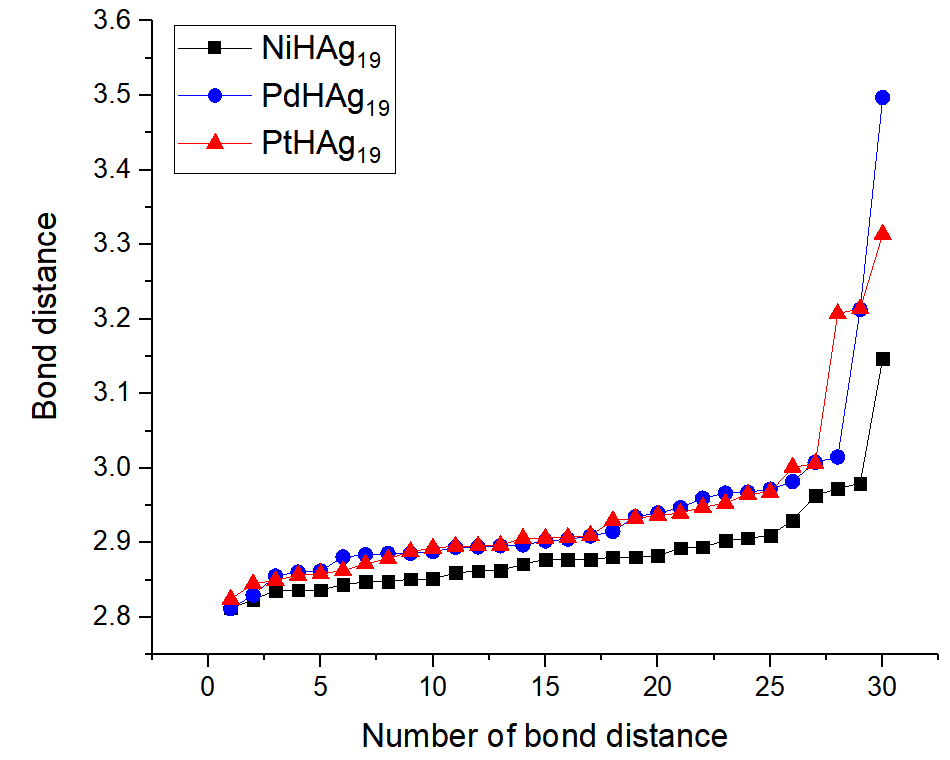


**Figure S9.** The distribution of Ag_ico_-Ag_ico_ distances in **NiHAg_19_** and MHAg_19_(dtp)_12_ (M = Pd, Pt).


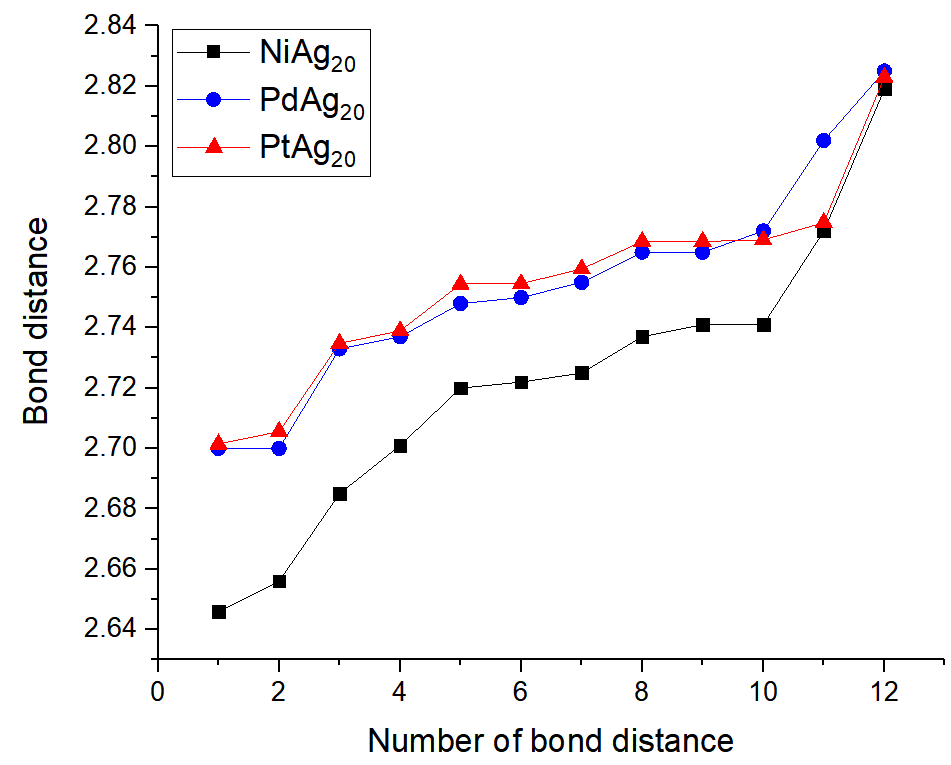


**Figure S10.** The distribution of Ni-Ag_ico_ distances in **NiAg_20_** and MAg_20_(dtp)_12_ (M = Pd, Pt).


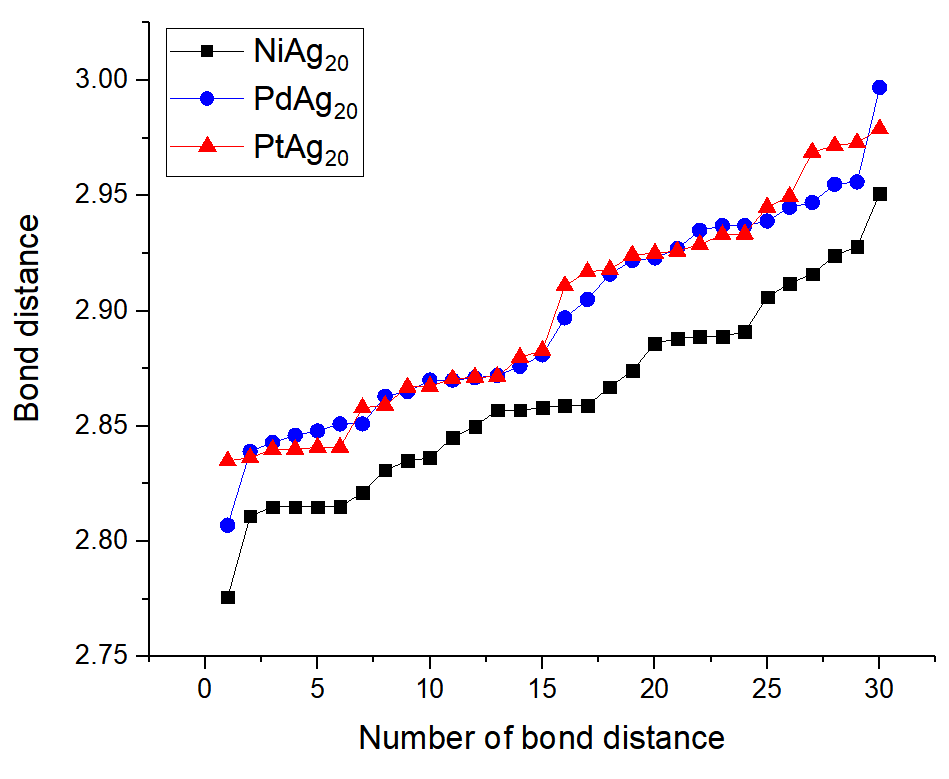


**Figure S11.** The distribution of Ag_ico_-Ag_ico_ distances in **NiAg_20_** and MAg_20_(dtp)_12_ (M = Pd, Pt).


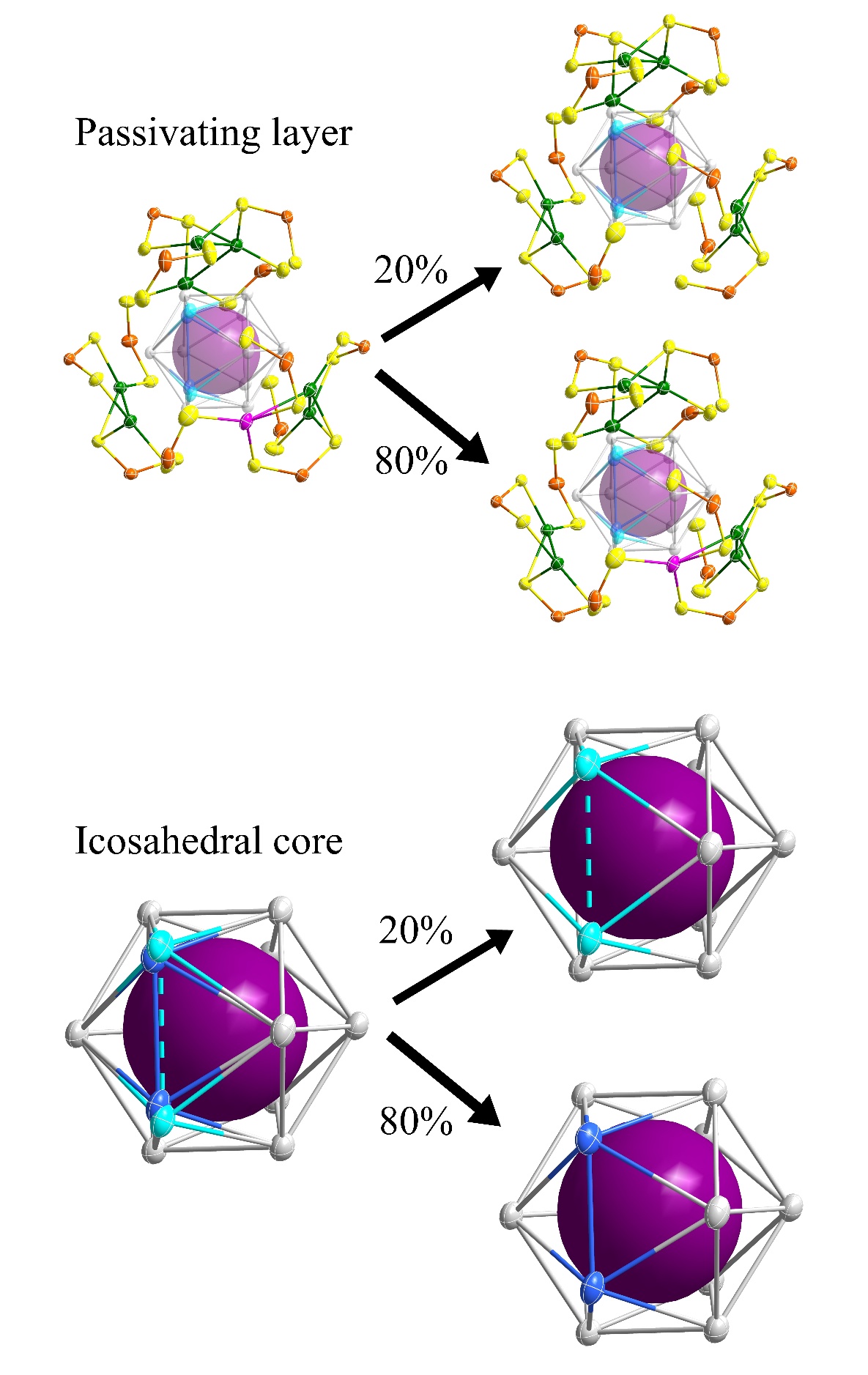


**Figure S12.** Passivating layer and icosahedral core of [**NiHAg_19_**]_0.2_[**NiAg_20_**]_0.8_

_
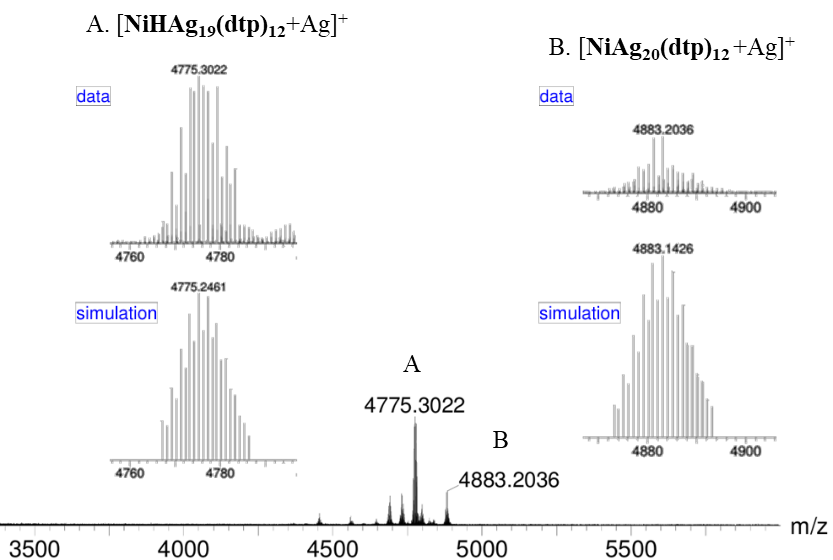
_

**Figure S13.** The positive-ion ESI-TOF-MS spectrum of the co-crystal. The experimental (top) and simulated (down) isotopic distributions are consistent.

_
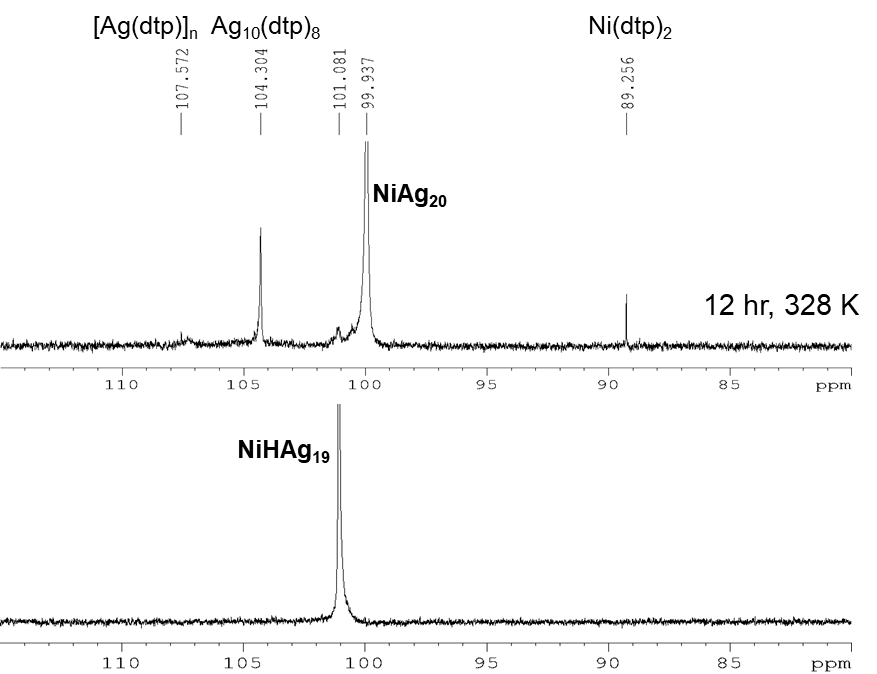
_

**Figure S14.** The ^31^P{^1^H} NMR spectra (202.46 MHz, CD_3_OD) of **NiHAg_19_** at ambient temperature and after heating for 12 hours_._

**
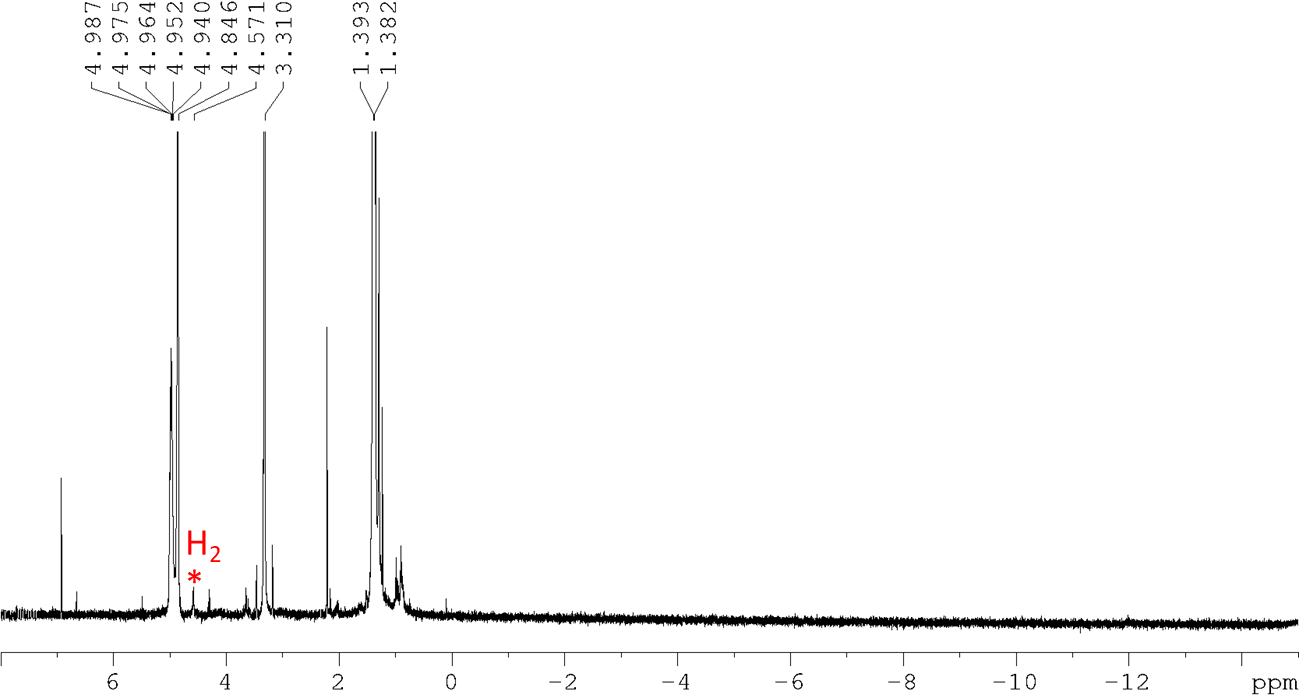
**

**Figure S15.** ^1^H NMR spectrum (500 MHz, CD_3_OD) of **NiHAg_19_**_._ The H_2_ gas is generated after heating for 12 hours.


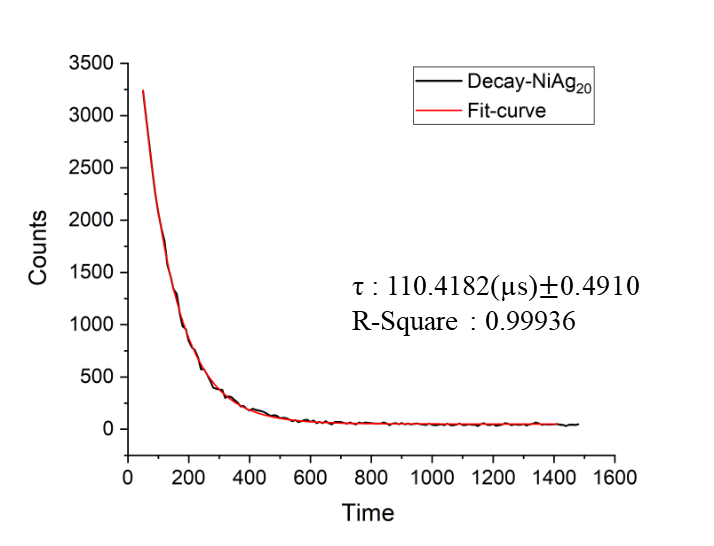


**Figure S16.** Time-resolved photoluminescence spectrum of **NiAg_20_** in 2Me-THF at 77 K.

**
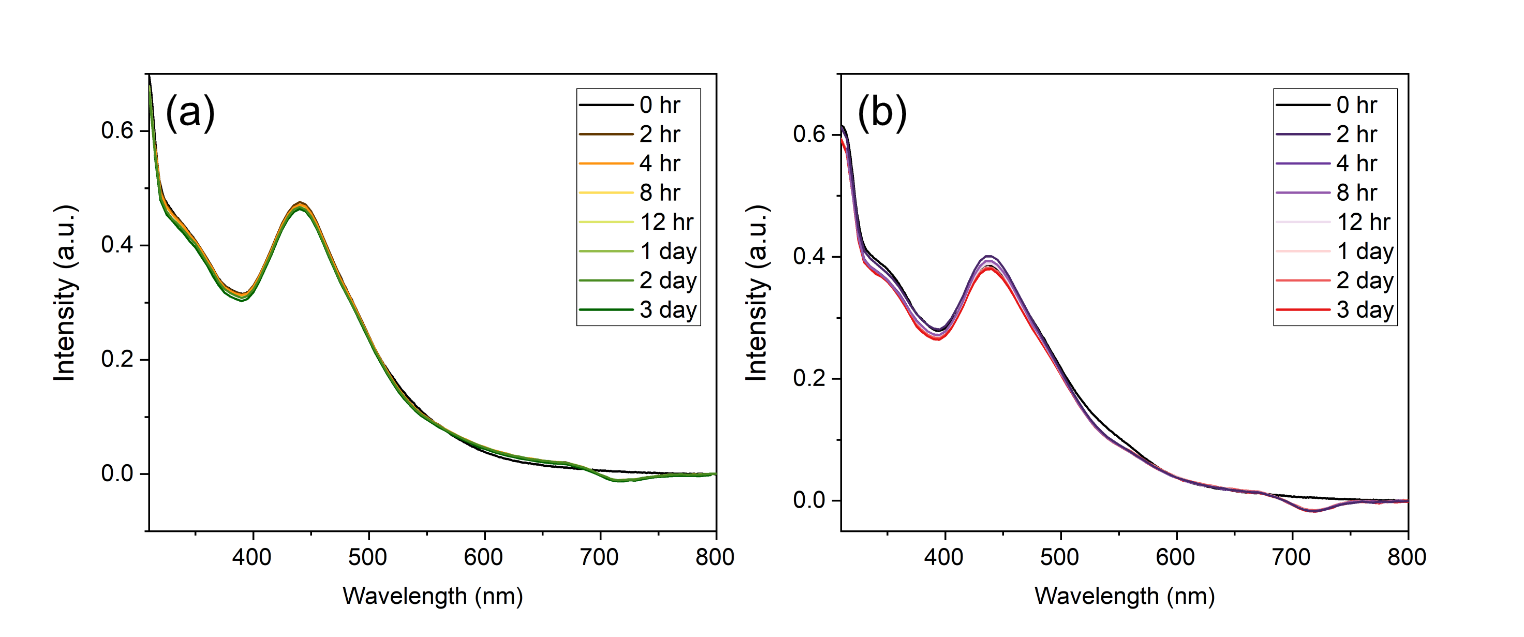
**

**Figure S17.** Time-dependent UV-vis spectra of **NiHAg_19_** in (a) DCM and (b) MeOH.

**
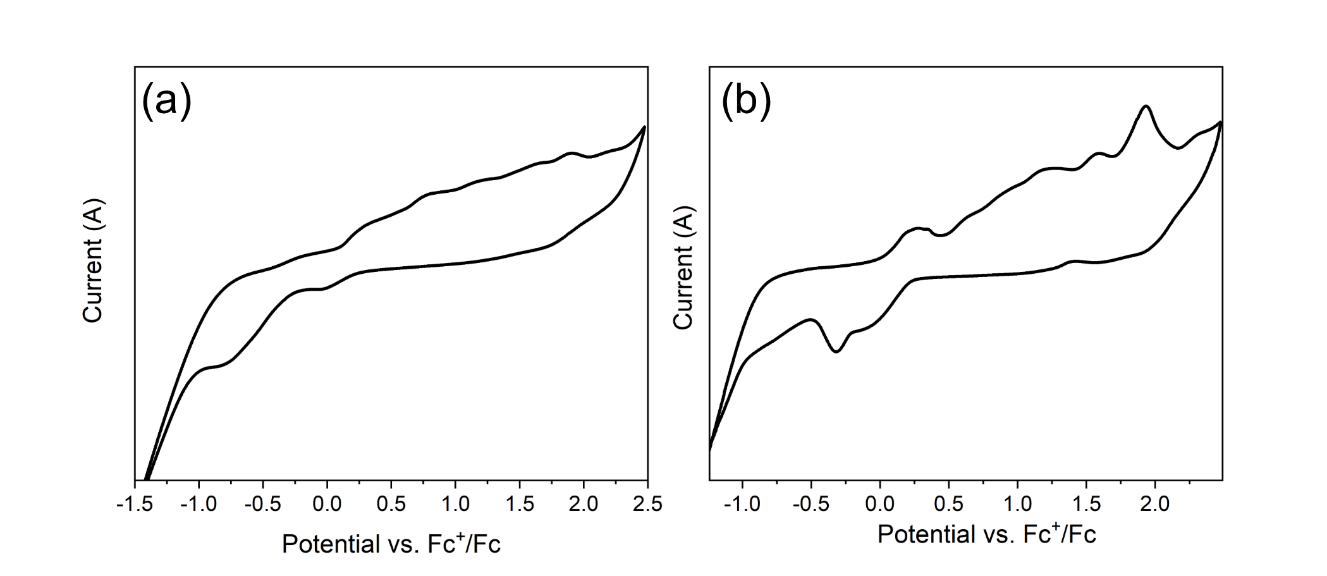
**

**Figure S18.** The cyclic voltammetry of (a) **NiHAg_19_** and (b) **NiAg_20_** in CH_2_Cl_2_ solution containing 0.1 M Bu_4_PF_6_ at 233 K.


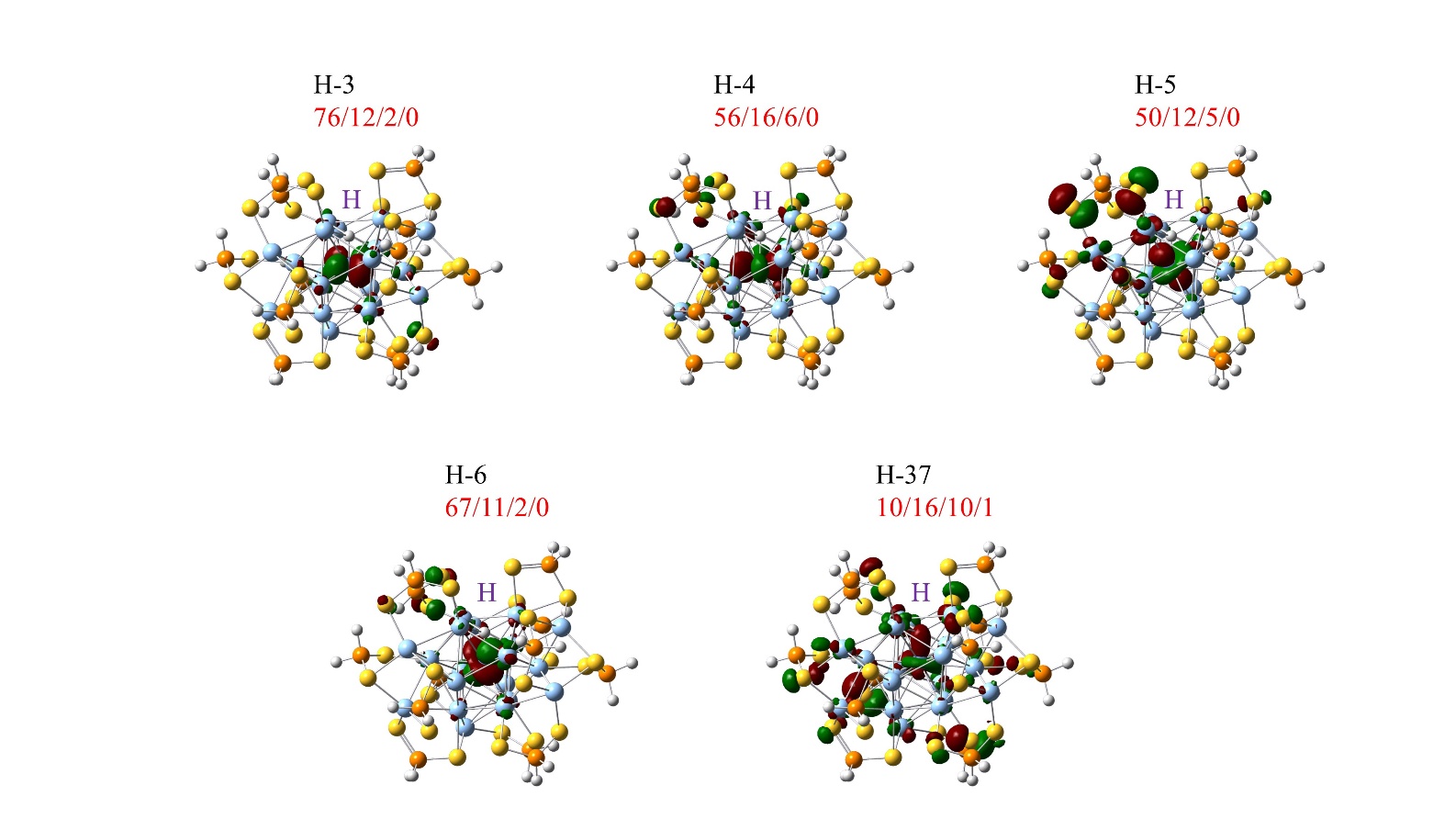


**Figure S19.** Kohn–Sham frontier orbital diagram of **NiHAg_19_** (HOMO-3 to HOMO-6 and HOMO-37). Values in red are the contribution (in %) of the core atoms or atom groups in the following order: Ni/Ag_ico_/Ag_cap_/H. (isovalue = 0.03)


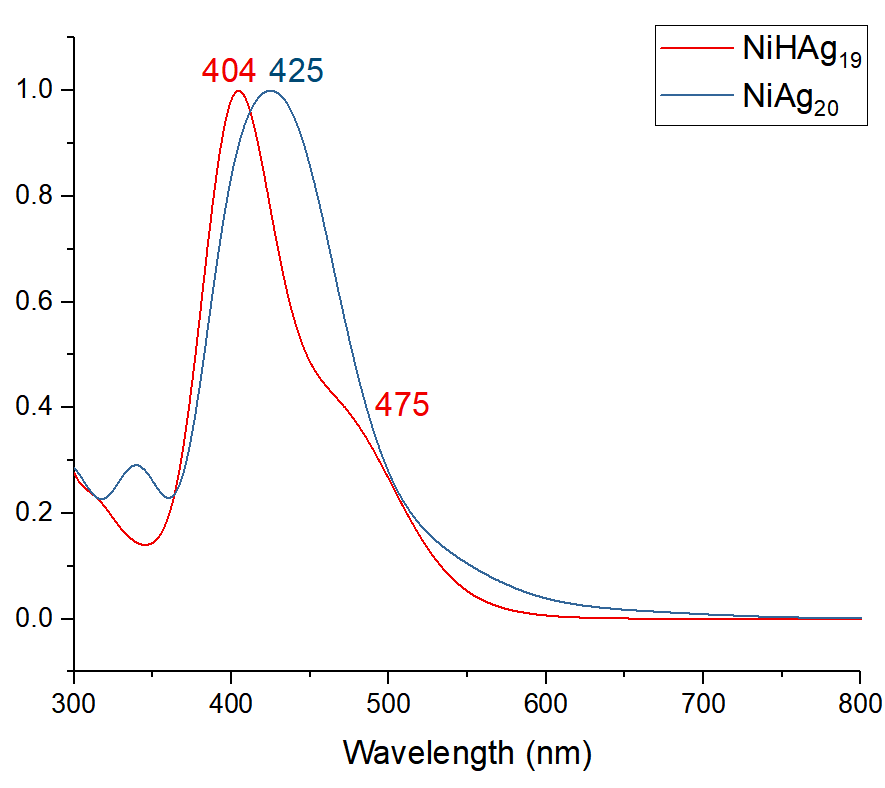


**Figure S20.** TD-DFT-simulated UV-vis absorption spectra of **NiHAg_19_** and **NiAg_20_**.


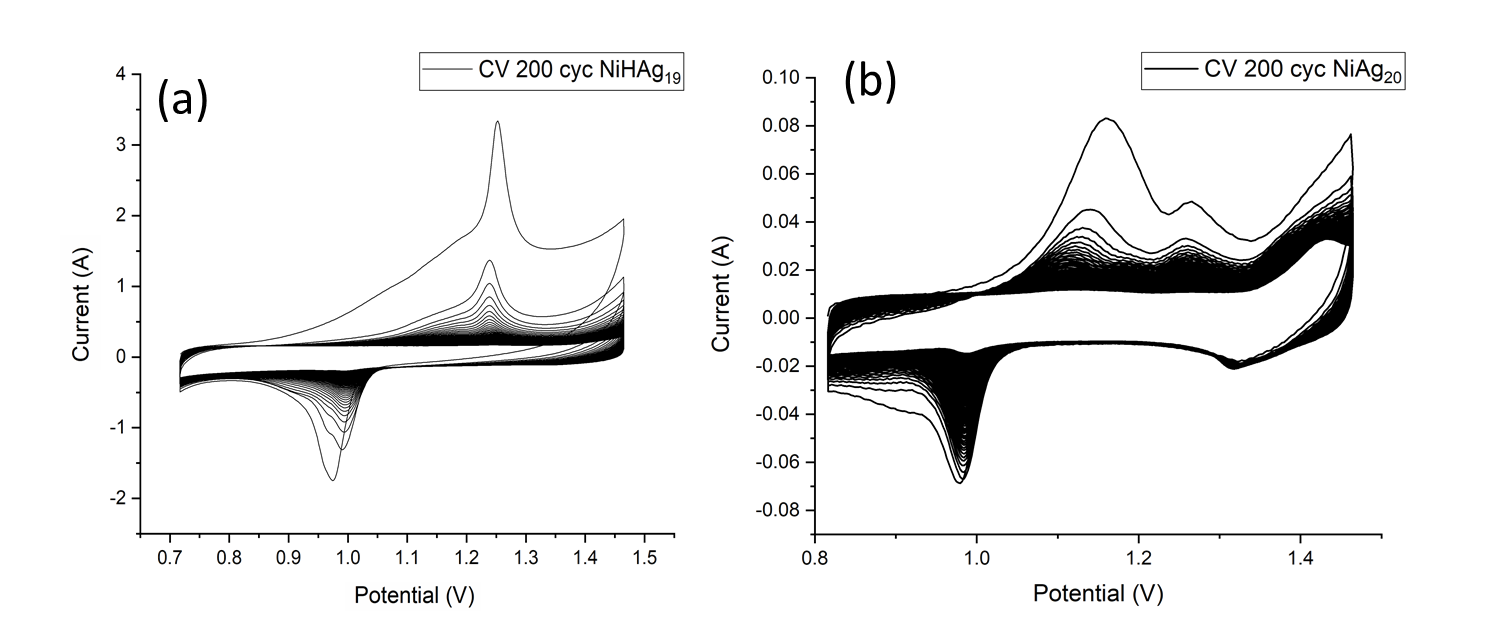


**Figure S21.** (a-b) CV activation of **NiHAg_19_** and **NiAg_20_** NCs by cycling at a sweep rate of 100 mV/s for 200 cycles.


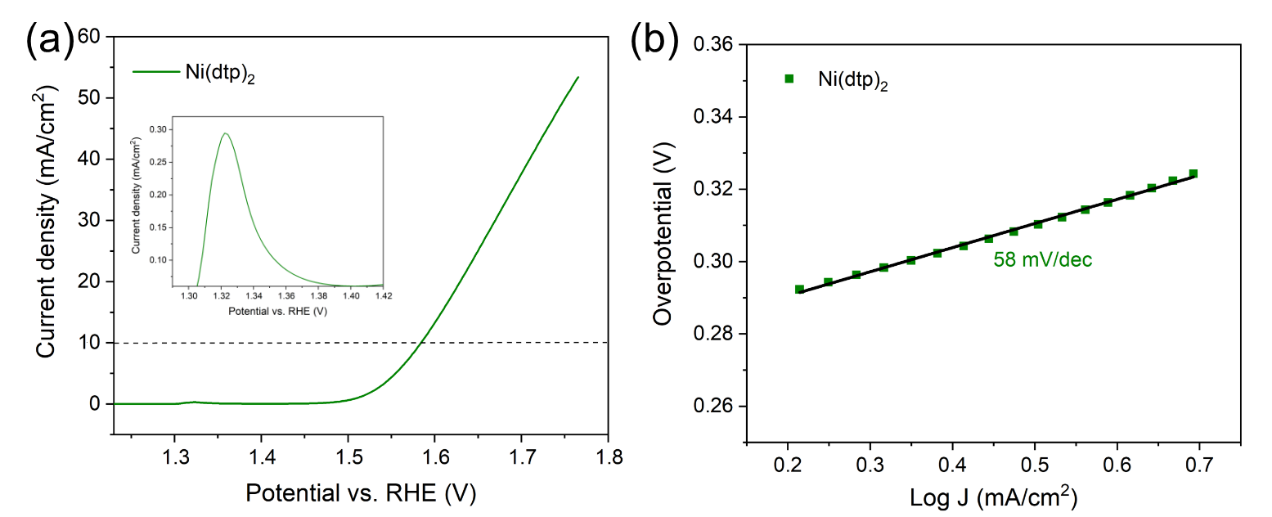


**Figure S22.** (a) LSV curve of Ni(dtp)_2_, and the inset of Ni^2+^/Ni^3+^ redox peak around 1.4 V. (b) Tafel slope of Ni(dtp)_2_.


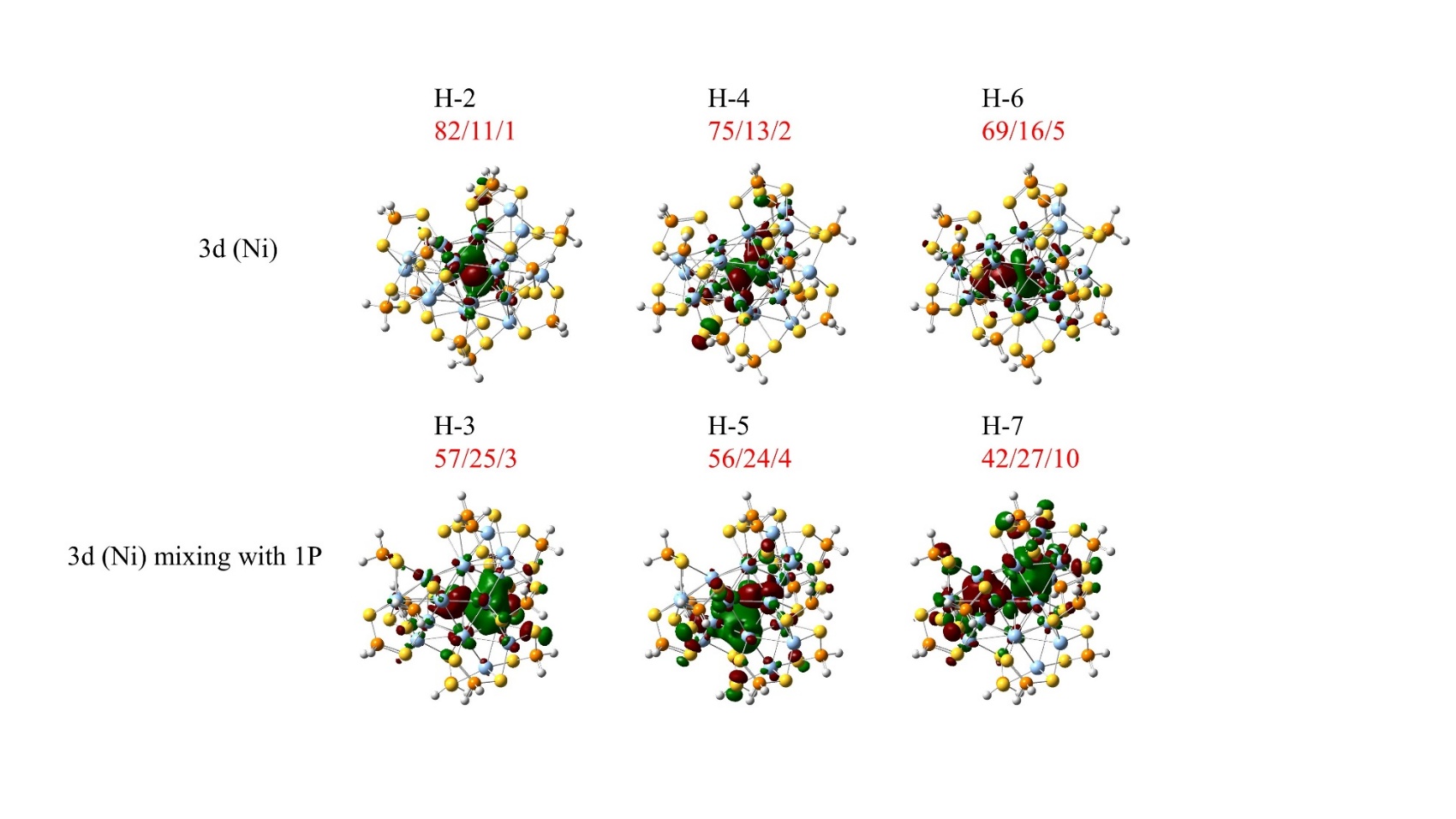


**Figure S23.** Kohn–Sham frontier orbital diagram of **NiAg_20_** (HOMO-2 to HOMO-7). Values in red are the contribution (in %) of the core atoms or atom groups in the following order: Ni/Ag_ico_/Ag_cap_. (isovalue = 0.02)

**Table S1.** Crystal data and refinement of **NiHAg_19_, [NiAg_20_]_0.8_[NiHAg_19_]_0.2_** and **NiAg_20_**.

| Compound | **NiHAg_19_** | **NiAg_20_** | **[NiAg_20_]_0.8_[NiHAg_19_]_0.2_** |
| --- | --- | --- | --- |
| Chemical formula | C_72_H_169_Ag_19_NiO_24_P_12_S_24_ | C_72_H_168_Ag_20_NiO_24_P_12_S_24_ | \| C_147_H_343_Ag_39.6_Ni_2_O_48_P_24_S_48_ \| \| --- \| \|  \| |
| CCDC | 2477724 | 2477725 | 2477726 |
| Formula weight | 4668.38 | 4775.24 | 9550.43 |
| Crystal system, space group | Triclinic, *P*$\bar{1}$ | Triclinic, *P*$\bar{1}$ | Triclinic, *P*$\bar{1}$ |
| a, Å | 15.7814(2) | 15.8988(4) | 15.8549(1) |
| b, Å | 18.1286(2) | \| 17.9715(5) \| \| --- \| \|  \| | 18.0359(1) |
| c, Å | 29.3581(3) | \| 29.3581(5) \| \| --- \| \|  \| | 29.3685(2) |
| α, deg. | 81.643(10) | \| 82.044(2) \| \| --- \| \|  \| | 81.843(1) |
| β, deg | 76.736(10) | \| 77.078(2) \| \| --- \| \|  \| | 76.944(1) |
| γ, deg | 64.819(10) | \| 65.956(3) \| \| --- \| \|  \| | 65.543(1) |
| Volume, Å3 | 7387.37(16) | \| 7456.0(4) \| \| --- \| \|  \| | 7435.9(1) |
| Z | 2 | 2 | 1 |
| ρcalcd, g·cm-3 | 2.099 | 2.127 | 2.133 |
| μ, mm-1 | 24.644 | \| 25.442 \| \| --- \| \|  \| | 25.309 |
| Temperature, K | 100.00(2) | 100.00(10) | 100.00(10) |
| θmax, deg. / Completeness, % | 67.080/99.4 | 69.999/98.7 | 69.995/98.7 |
| Reflections collected / unique | 310786/26251 [R_int_ = 0.0851] | 120653/27941 [R_int_ = 0.0903] | 131791/27847  [R_int_ = 0.0415] |
| Restraints/parameters | 568/1479 | 456/1437 | 564/1526 |
| R1a, wR2b [I > 2σ(I)] | R_1_ = 0.0454, wR_2_ = 0.1226 | R_1_ = 0.0782, wR_2_ = 0.2119 | R_1_ = 0.0379, wR_2_ = 0.0956 |
| R1a, wR2b (all data) | R_1_ = 0.0481, wR_2_ = 0.1247 | R_1_ = 0.0936, wR_2_ = 0.2264 | R_1_ = 0.0418, wR_2_ = 0.0980 |
| Goodness of fit | 1.018 | 1.040 | 1.015 |
| Largest diff. peak and hole, e/Å3 | 2.32/-1.71 | 3.44/-2.92 | 1.55/-1.87 |

^a^*R*1 = Σ｜︱*F_o_*︱-︱*F_c_*︱｜/ Σ︱*F_o_*︱. *^b^wR*2 = { Σ[*w*(*F_o_*^2^ - F_c_^2^)^2^] / Σ[*w*(*F*_o_^2^)_2_] }^1/2^

**Table S2.** Atomic coordinates of the DFT-optimized structure of **NiHAg_19_** and **NiAg_20_**

**NiHAg_19_**

Ag -0.139000 -2.448000 1.253000

Ag 2.273000 -1.225000 0.121000

Ag 1.175000 -0.146000 2.643000

Ag -2.051000 -0.476000 2.449000

Ag -2.501000 -1.490000 -0.299000

Ag -0.002000 -2.041000 -1.654000

Ag 1.406000 0.467000 -2.122000

Ag 2.144000 1.684000 0.518000

Ag -0.413000 2.350000 1.869000

Ag -2.641000 1.411000 0.138000

Ag -0.167000 2.648000 -1.025000

Ag -1.521000 0.394000 -2.340000

Ag 2.593000 -2.928000 2.678000

Ag 1.695000 -4.247000 -0.408000

Ag 3.010000 -2.255000 -2.647000

Ag 2.785000 3.220000 -2.058000

Ag 1.308000 4.729000 0.747000

Ag -4.948000 -0.342000 1.415000

Ag -4.616000 0.183000 -1.957000

P 4.044000 -0.112000 4.480000

P -0.033000 -5.485000 2.547000

P 5.244000 -3.305000 0.316000

P -4.004000 -0.950000 4.885000

P 0.721000 4.433000 4.300000

P 4.738000 4.140000 0.898000

P 3.954000 0.771000 -4.445000

P 0.786000 -4.819000 -3.932000

P -5.790000 -2.955000 -0.937000

P -5.556000 2.993000 0.047000

P -3.115000 0.093000 -5.210000

P 0.123000 5.573000 -2.578000

S 2.104000 0.249000 4.939000

S 4.638000 -1.943000 3.871000

S 0.332000 -3.818000 3.644000

S -0.485000 -5.291000 0.586000

S 3.881000 -4.637000 1.021000

S 4.747000 -1.642000 -0.752000

S -2.761000 0.609000 4.793000

S -4.214000 -2.075000 3.183000

S -1.135000 3.927000 3.676000

S 1.956000 5.496000 3.108000

S 4.634000 2.137000 1.032000

S 3.653000 5.152000 -0.508000

S 4.624000 1.541000 -2.720000

S 2.342000 -0.491000 -4.433000

S 2.524000 -4.794000 -2.840000

S -0.612000 -3.400000 -3.760000

S -6.477000 -1.056000 -0.572000

S -4.012000 -3.588000 -0.273000

S -4.724000 2.240000 1.745000

S -4.562000 2.755000 -1.696000

S -1.928000 1.598000 -4.621000

S -3.696000 -1.296000 -3.841000

S -0.756000 5.307000 -0.766000

S 0.811000 3.958000 -3.576000

H -0.496000 0.354000 1.686000

H 3.639000 1.774000 -5.400000

H 5.028000 0.106000 -5.095000

H 6.100000 4.490000 0.696000

H 4.448000 4.752000 2.147000

H 1.133000 6.566000 -2.470000

H -0.817000 6.230000 -3.415000

H -4.260000 0.610000 -5.878000

H -2.520000 -0.645000 -6.268000

H -5.921000 -3.141000 -2.339000

H -6.790000 -3.838000 -0.449000

H -6.900000 2.544000 -0.066000

H -5.754000 4.384000 0.255000

H 1.394000 3.275000 4.777000

H 0.557000 5.170000 5.503000

H -5.293000 -0.565000 5.350000

H -3.641000 -1.875000 5.902000

H -1.107000 -6.175000 3.168000

H 1.026000 -6.415000 2.724000

H 0.229000 -6.107000 -3.708000

H 1.181000 -4.940000 -5.291000

H 6.193000 -4.040000 -0.440000

H 6.012000 -2.850000 1.418000

H 4.501000 0.872000 3.560000

H 4.800000 0.214000 5.637000

Ni -0.170000 0.090000 0.161000

**NiAg_20_**

Ni -0.004000 -0.021000 0.012000

Ag 1.656000 -1.313000 -1.811000

Ag 1.505000 1.604000 -1.698000

Ag 2.681000 0.088000 0.556000

Ag 1.155000 -2.405000 0.902000

Ag -1.006000 -2.402000 -1.097000

Ag -0.739000 0.046000 -2.749000

Ag -1.185000 2.358000 -0.926000

Ag 0.926000 2.350000 1.146000

Ag 0.805000 -0.118000 2.763000

Ag -1.498000 -1.621000 1.734000

Ag -2.683000 -0.086000 -0.546000

Ag -1.639000 1.287000 1.849000

Ag 1.109000 4.603000 -0.838000

Ag -1.495000 4.318000 1.365000

Ag -3.288000 -1.751000 -3.065000

Ag -3.747000 -2.921000 0.124000

Ag 4.290000 0.380000 -1.981000

Ag 3.995000 -2.607000 -0.406000

Ag 3.578000 -1.820000 2.737000

Ag -4.384000 -0.300000 1.937000

S 2.983000 -3.510000 -2.661000

S 3.628000 -0.487000 -4.326000

S 1.953000 3.802000 -3.159000

S 4.826000 2.845000 -1.371000

S 4.822000 0.420000 2.134000

S 6.168000 -1.104000 -0.801000

S 4.686000 -3.986000 1.712000

S 1.381000 -4.985000 0.994000

S -1.683000 -3.734000 -3.553000

S -2.218000 -4.992000 -0.347000

S -0.309000 -0.230000 -5.319000

S -3.512000 0.539000 -4.190000

S -2.273000 3.950000 -2.655000

S -0.849000 6.241000 -0.353000

S 2.667000 4.397000 1.293000

S -0.050000 4.398000 3.518000

S 1.568000 1.046000 4.962000

S 2.069000 -2.400000 4.719000

S -2.140000 -2.708000 3.997000

S -5.241000 -2.729000 2.283000

S -5.216000 0.752000 -0.367000

S -5.513000 -2.498000 -1.834000

S -3.515000 1.362000 3.733000

S -4.055000 3.879000 1.323000

P 3.972000 -2.454000 -4.096000

P 3.980000 3.812000 -2.910000

P 6.389000 -0.123000 0.961000

P 3.076000 -5.218000 2.027000

P -2.399000 -5.199000 -2.350000

P -2.179000 0.362000 -5.718000

P -2.186000 5.770000 -1.834000

P 1.584000 5.300000 2.770000

P 2.110000 -0.688000 5.809000

P -3.973000 -3.447000 3.730000

P -6.270000 -0.674000 -1.361000

P -4.331000 3.152000 3.168000

H 3.692000 -3.093000 -5.332000

H 5.363000 -2.719000 -3.967000

H 4.519000 3.387000 -4.153000

H 4.415000 5.163000 -2.874000

H -1.998000 6.737000 -2.858000

H -3.453000 6.166000 -1.328000

H -2.189000 1.626000 -6.370000

H -2.740000 -0.450000 -6.743000

H -6.735000 -0.076000 -2.561000

H -7.488000 -0.895000 -0.667000

H -1.752000 -6.409000 -2.712000

H -3.745000 -5.501000 -2.695000

H 3.552000 -6.542000 1.832000

H 2.843000 -5.217000 3.429000

H 7.305000 -0.868000 1.748000

H 7.135000 1.057000 0.706000

H 1.292000 6.631000 2.364000

H 2.483000 5.539000 3.843000

H -5.728000 3.090000 3.419000

H -3.918000 4.076000 4.165000

H -3.943000 -4.853000 3.528000

H -4.697000 -3.373000 4.950000

H 1.316000 -0.954000 6.959000

H 3.387000 -0.545000 6.421000

References

[53] A. A. M. Aly, B.Walfort, H. Lang, *Z. Kristallogr.- New Cryst. Struct.* **2004**, *219*, 489.

[54] P. Wystrach, E. O. Hook, G. L. M. Christopher, *J. Org. Chem.* **1956**, *21*, 705.

[55] P. J. H. A. M. van de Leempert, T. W. Hummelink, J. H. Noordik, P. T. Beurskens, *Cryst. Struct. Commun.* **1975**, *4*, 167.

[56] L. J. Bourhis, O. V. Dolomanov, R. J. Gildea, J. A. K. Howard, H. Puschmann, *Acta Crystallogr., Sect. A: Found. Crystallogr.* **2015**, *71*, 59.

[57] O. V. Dolomanov, L. J. Bourhis, R. J. Gildea, J. A. K. Howard, H. Puschmann, *J. Appl. Crystallogr.* **2009**, *42*, 339.

[58] M. J. Frisch, G. W. Trucks, H. B. Schlegel, G. E. Scuseria, M. A. Robb, J. R. Cheeseman, G. Scalmani, V. Barone, G. A. Petersson, H. Nakatsuji, X. Li, M. Caricato, A. V. Marenich, J. Bloino, B. G. Janesko, R.Gomperts, B.Mennucci, H. P. Hratchian, J. V. Ortiz, A. F. Izmaylov, J. L. Sonnenberg, D. Williams-Young, F. Ding, F. Lipparini, F. Egidi, J. Goings, B. Peng, A. Petrone, T. Henderson, D. Ranasinghe, et al., *Gaussian 16, Revision C.01*, Gaussian, Inc, Wallingford CT **2016**.

[59] A. D. Becke, *Phys. Rev. A* **1988**, *38*, 3098.

[60] J. P. Perdew, *Phys. Rev. B* **1986**, *33*, 8822.

[61] A. Schäfer, H. Horn, R. Ahlrichs, *J. Chem. Phys.* **1992**, *97*, 2571.

[62] A. Schäfer, C. Huber, R. Ahlrichs, *J. Chem. Phys.* **1994**, *100*, 5829.

[63] S. I. Gorelsky, *AOMix program for molecular orbital analysis*, version 6.35; University of Ottawa:, Ottawa, Canada, **2007**.

[64] E. D. Glendening, C. R. Landis, F. Weinhold, *J. Comput. Chem.* **2013**, *34*, 1429.

[65] T. Yanai, D. P. Tew, N. C. Handy, *Chem. Phys. Lett.* **2004**, *393*, 51.

[66] S. I. Gorelsky, A. B. P. Lever, *J. Organomet. Chem.* **2001**, *635*, 187.
